# Stbd1 promotes glycogen clustering during endoplasmic reticulum stress and supports survival of mouse myoblasts

**DOI:** 10.1242/jcs.244855

**Published:** 2020-10-26

**Authors:** Andria A. Lytridou, Anthi Demetriadou, Melina Christou, Louiza Potamiti, Nikolas P. Mastroyiannopoulos, Kyriacos Kyriacou, Leonidas A. Phylactou, Anthi Drousiotou, Petros P. Petrou

**Affiliations:** 1 Department of Biochemical Genetics, The Cyprus Institute of Neurology and Genetics, P.O. Box 23462, 1683 Nicosia, Cyprus; 2 Department of Molecular Genetics, Function and Therapy, The Cyprus Institute of Neurology and Genetics, P.O. Box 23462, 1683 Nicosia, Cyprus; 3 Department of Electron Microscopy/Molecular Pathology, The Cyprus Institute of Neurology and Genetics, P.O. Box 23462, 1683 Nicosia, Cyprus; 4 Cyprus School of Molecular Medicine, P.O. Box 23462, 1683 Nicosia, Cyprus

**Keywords:** Glycogen, ER stress, Glycogenin, Glycogen synthase, UPR, Apoptosis

## Abstract

Imbalances in endoplasmic reticulum (ER) homeostasis provoke a condition known as ER stress and activate the unfolded protein response (UPR) pathway, an evolutionarily conserved cell survival mechanism. Here, we show that mouse myoblasts respond to UPR activation by stimulating glycogenesis and the formation of α-amylase-degradable, glycogen-containing ER structures. We demonstrate that the glycogen-binding protein Stbd1 is markedly upregulated through the PERK signalling branch of the UPR pathway and is required for the build-up of glycogen structures in response to ER stress activation. In the absence of ER stress, Stbd1 overexpression is sufficient to induce glycogen clustering but does not stimulate glycogenesis. Glycogen structures induced by ER stress are degraded under conditions of glucose restriction through a process that does not depend on autophagosome–lysosome fusion. Furthermore, we provide evidence that failure to induce glycogen clustering during ER stress is associated with enhanced activation of the apoptotic pathway. Our results reveal a so far unknown response of mouse myoblasts to ER stress and uncover a novel specific function of Stbd1 in this process, which may have physiological implications during myogenic differentiation.

This article has an associated First Person interview with the first author of the paper.

## Abstract (precis)

**Highlighted Article:** ER stress in mouse myoblasts activates a cell survival mechanism that involves the upregulation of Stbd1 expression that is required for the formation of glycogen-containing structures.

## INTRODUCTION

The endoplasmic reticulum (ER) is an intracellular network of interconnected membranes involved in a variety of cellular processes including synthesis, folding and maturation of secretory and transmembrane proteins (Schwarz and Blower, 2016). A balance between protein load and folding capacity of the ER ensures proper protein folding, processing and quality control. This balance can be disrupted by physiological or pathological stimuli, resulting in the accumulation of misfolded proteins, a condition described as ER stress. An evolutionarily conserved adaptive mechanism integrating specific signalling pathways, known as the unfolded protein response (UPR), is activated by ER stress and primarily aims to restore ER homeostasis. UPR induction triggers a series of cellular events, such as attenuation of global protein translation and activation of specific signalling mechanisms, which promote cell adaptation and survival. However, in the case of unresolved ER stress, the UPR triggers cell death by apoptosis (Ron and Walter, 2007; Hotamisligil, 2010; Hetz, 2012).

In mammals, the UPR is mediated by three transmembrane ER proteins: IRE1 (also known as ERN1), ATF6 and PERK (also known as EIF2AK3), which function as sensors continuously monitoring the ER environment. Under steady-state conditions, the luminal domains of these sensors interact with the ER chaperone BiP (HSPA5) and are thus held inactive. Accumulation of misfolded proteins in the ER lumen causes the detachment of BiP from the above sensors, which become activated and initiate distinct signalling pathways, known as the UPR branches (Hetz, 2012).

ER stress and UPR activation influence various cellular processes including pathways of glucose, lipid and glycogen metabolism (Hotamisligil, 2010). Glycogen is a large, multi-branched glucose polymer which serves as a readily mobilized storage form of energy, primarily in liver and muscle (Prats et al., 2018). Glycogen is present in the form of granules consisting of carbohydrate and proteins that vary between tissues. The electron microscopic appearance of glycogen suggests the existence of two main structures, termed α- and β-particles. α-particles are the larger of the two structures, are found mainly in the liver and result from the association of several smaller β-particles. Glycogen is stored as β-particles in muscle and other tissues, such as the brain. A β-particle corresponds to a single glycogen unit and its synthesis begins with the dimerization and autoglycosylation of the central priming protein, glycogenin (GN). Glucose polymerization and branching is achieved through the coordinated action of glycogen synthase and the branching enzyme. Under conditions of glucose shortage, glycogen granules are degraded through the combined action of glycogen phosphorylase and the debranching enzyme in the cytosol, or alternatively by acid α-glucosidase in lysosomes.

It has been previously reported that ER stress activation in neuronal cells induces the formation of polyglucosan, a poorly branched form of glycogen, through the activation of glycogen synthase 1 (GS1, also known as GYS1), the main glucose polymerizing enzyme in tissues other than the liver (Wang et al., 2013). Although the formation of polyglucosan in a mouse model of Lafora disease (OMIM #254780) was associated with accelerated disease onset and neurodegeneration, it has also been proposed to serve the rapid capture and supply of energy to support neuronal cell survival during ER stress (Wang et al., 2013).

In the present study, we report that the starch binding domain-containing protein 1 (Stbd1) links ER stress with the build-up of glycogen structures in mouse myoblasts. Stbd1 is a transmembrane, glycogen-binding, ER-resident protein, also found at ER–mitochondria contact sites, at which it is implicated in ER–mitochondria tethering (Jiang et al., 2010; Demetriadou et al., 2017). Based on the presence of an Atg8-family interacting motif (AIM), mediating its interaction with the autophagy protein Gabarapl1, Stbd1 is thought to function as a selective autophagy receptor for glycogen (Jiang et al., 2011). However, definitive evidence for the above is currently lacking, and the role of Stbd1 in glycogen metabolism remains largely uncharacterized.

Here, we show that Stbd1 is markedly upregulated in response to ER stress activation and is both necessary and sufficient to promote glycogen clustering in mouse myoblasts, in a process that involves the recruitment of GN and GS1 to the ER membrane. Our results indicate that Stbd1 has a specific role during ER stress and is not required for the formation of glycogen clusters induced by overexpression of the protein phosphatase 1 subunit protein targeting to glycogen (PTG, also known as PPP1R3C). We demonstrate that ER stress-induced glycogen structures are degraded under conditions of glucose restriction through a mechanism that does not depend on autophagosome–lysosome fusion. Furthermore, our results indicate that deficiency in the formation of glycogen structures in response to ER stress is associated with enhanced susceptibility to apoptosis.

## RESULTS

### ER stress activation induces glycogen synthesis and clustering in mouse myoblasts

We have previously reported that, under standard culturing conditions, C2C12 mouse myoblasts sporadically display glycogen structures, which colocalize with Stbd1 and the ER membrane marker calnexin (Demetriadou et al., 2017). We reasoned that the formation of these structures in only very few but not the bulk of cells occurs in response to a specific stimulus and sought to identify the event triggering their build-up.

**Fig. 1.**
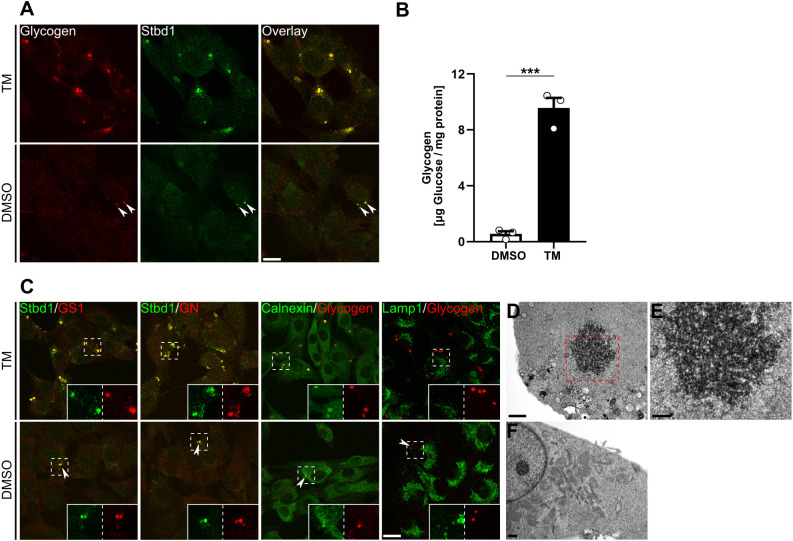
**ER stress activation induces glycogenesis and the formation of glycogen-containing ER structures.** (A) Representative immunofluorescence images of C2C12 myoblasts treated with TM or DMSO (control) and stained for glycogen and Stbd1. TM-treated cells display prominent glycogen structures that strongly coincide with Stbd1 (overlay) [mean±s.e.m. Manders’ colocalization coefficient (MCC), 0.746±0.032; *n*=10]. DMSO-treated controls occasionally display smaller Stbd1-positive glycogen structures (arrowheads) (MCC, 0.615±0.025; *n*=6). (B) Glycogen quantification in DMSO- and TM-treated C2C12 myoblasts revealed that ER stress activation stimulates glycogen synthesis (mean±s.e.m. μg glucose/mg protein: DMSO-treated, 0.55±0.20; TM-treated, 9.56±0.74; *n*=3). ***P≤0.001 (one-tailed unpaired Student's *t*-test). (C) Representative images of TM- and DMSO-treated C2C12 myoblasts immunostained with the indicated antibodies. In addition to positive Stbd1 immunostaining, both the ER stress-induced glycogen structures and those sporadically present in DMSO-treated controls (arrowheads) stain positive for GS1 (MCC: TM-treated, 0.785±0.012, *n*=10; DMSO-treated, 0.565±0.052, *n*=7), GN (MCC: TM-treated, 0.791±0.011, *n*=10; DMSO-treated, 0.703±0.025, *n*=8) as well as calnexin (MCC: TM-treated, 0.752±0.019, *n*=10; DMSO-treated, 0.763±0.040, *n*=7) but not Lamp1 (MCC: TM-treated, 0.047±0.010, *n*=10; DMSO-treated, 0.028±0.009, *n*=10). Inserts show single stainings at higher magnification for the corresponding areas indicated by dashed boxes. (D–F) Transmission electron micrographs of TM- (D,E) and DMSO- treated (F) C2C12 cells. TM-treated cells display prominent membrane-free glycogen structures not present in DMSO-treated control cells. A higher magnification image of the boxed area in D is shown in E. Scale bars: 20 μm (A,C), 2 μm (D,F), 1 μm (E).

We found that treatment of C2C12 myoblasts with the pharmacological ER stress inducer tunicamycin (TM) for 16 h resulted in the formation of prominent glycogen structures in the majority of cells (Fig. 1A). This was not the case for control cells treated with DMSO (vehicle), in which only small glycogen structures were occasionally detected. Importantly, both the ER stress-induced glycogen clusters and those present in control cells stained positive for Stbd1, suggesting its potential implication in their formation (Fig. 1A).

To glean insights into the molecular mechanisms driving the ER stress-induced formation of glycogen clusters in C2C12 myoblasts, we first addressed the question of whether ER stress activation stimulates glycogen synthesis. For this, we quantified glycogen levels in cells treated with TM and in DMSO-treated controls. This revealed that TM-treated cells displayed significantly increased glycogen levels (Fig. 1B), suggesting that ER stress induction in C2C12 cells stimulates glycogenesis.

Human Stbd1 was previously reported to interact *in vitro* with glycogen synthase (Zhu et al., 2014). Moreover, in a recent proteomic study, human Stbd1 was suggested to interact with additional proteins implicated in glycogen metabolism, such as GN (Huttlin et al., 2017). Given the above, we evaluated whether the muscle-specific glycogen synthase (GS1) and GN were colocalized with Stbd1 on ER stress-induced glycogen clusters in C2C12 myoblasts. Indeed, in TM-treated cells both GS1 and GN coincided with Stbd1 on glycogen structures (Fig. 1C). Furthermore, these structures stained positive for calnexin, suggesting the involvement of the ER membrane in their formation. Similarly, GS1, GN and calnexin were detected on Stbd1-positive glycogen clusters, which were sparsely detectable in DMSO-treated controls (Fig. 1C, arrowheads). Importantly, glycogen structures induced either by TM treatment or present in DMSO-treated cells did not colocalize with Lamp1, suggesting that they are not associated with late endosomes or lysosomes and are likely cytosolic (Fig. 1C).

**Fig. 2.**
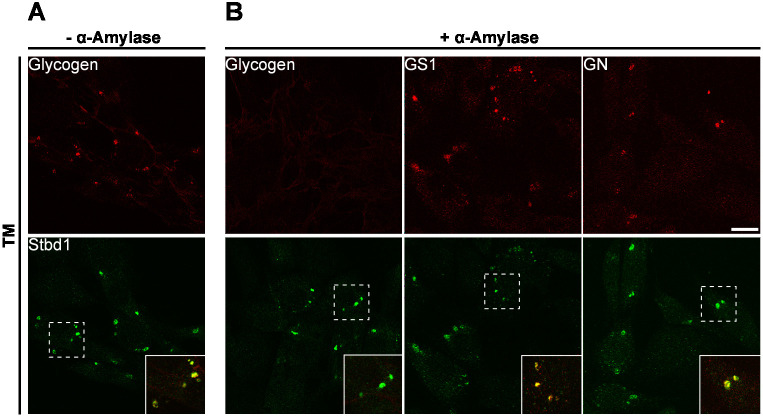
**ER stress-induced structures contain α-amylase-degradable glycogen.** TM-treated C2C12 cells were incubated either in the absence (A) or presence (B) of α-amylase and double-stained for Stbd1 (green) and glycogen, GS1 or GN (red). α-amylase treatment resulted in glycogen depletion from the ER stress-induced structures, which still displayed positive immunostaining for Stbd1 [mean±s.e.m. Manders’ colocalization coefficient (MCC): −α-amylase, 0.675±0.008, *n*=10; +α-amylase, 0.022±0.006, *n*=10], GS1 (MCC: 0.661±0.031, *n*=10) and GN (MCC: 0.528±0.025, *n*=10). Overlays are shown as inserts and represent higher magnifications of the indicated boxed areas. Representative images are shown. Scale bar: 20 μm.

Comparable results were obtained using additional ER stressors such as 2-deoxy-D-glucose (2-DG) and thapsigargin (TG) (Fig. S1A). This suggests that formation of glycogen structures encompassing Stbd1, GS1 and GN in C2C12 myoblasts occurs as a general response to ER stress, irrespective of the molecular mechanism disrupting ER homeostasis. TM and TG induced the formation of a similar number of structures per cell, which appeared to be increased when compared to the number of structures induced by 2-DG (Fig. S1B). Intriguingly, whereas glycogen structures induced by TM (Fig. 1C) and 2-DG (Fig. S1) were uniformly stained with calnexin, TG treatment resulted in the formation of both calnexin-positive (Fig. S1A, filled arrowheads) and calnexin-negative (Fig. S1A, open arrowheads) glycogen clusters.

To further characterize glycogen structures induced by ER stress, we performed transmission electron microscopy on C2C12 myoblasts treated either with TM or with DMSO as a control. At the ultrastructural level, TM-treated cells displayed large intracytoplasmic glycogen structures that were not membrane enclosed (Fig. 1D,E). Comparable structures were not present in control cells (Fig. 1F).

We next set out to validate the aforementioned findings in cultures of satellite cell-derived mouse primary myoblasts. For this, primary myoblasts were treated with TM, or DMSO as a control, and immunostained for glycogen, Stbd1, GS1 and GN. As observed in C2C12 cells, ER stress induction in primary myoblasts resulted in the formation of profound glycogen clusters (Fig. S2). Moreover, these stained positive for Stbd1, GS1 and GN, in agreement with the findings described for C2C12 cells. This suggests that the build-up of glycogen structures following TM treatment is not a cell-line specific event, because both C2C12 and primary myoblasts exhibited the same cellular response to ER stress activation. Based on the above and because of the ease of handling, C2C12 myoblasts were employed for subsequent experiments in this study.

Taken together, the above findings suggest that ER stress induction by TM in mouse myoblasts stimulates glycogenesis and further results in glycogen accumulation on discernible ER structures, on which Stbd1, GS1 and GN are localized. Moreover, the presence of the above proteins on glycogen structures occasionally observed in C2C12 cells under steady-state conditions suggests that formation of these structures occurs through the same molecular events. C2C12 myoblasts harboring such glycogen clusters under normal conditions could therefore represent individual cells undergoing ER stress.

### ER stress-induced glycogen-containing structures consist of α-amylase-degradable glycogen

It has been reported previously that ER stress induction in Neuro2A (N2A) neuroblastoma cells results in the formation of polyglucosan, a poorly branched form of glycogen that is resistant to α-amylase degradation (Wang et al., 2013). To evaluate whether glycogen clusters formed in response to ER stress in C2C12 myoblasts consist of normally structured glycogen or polyglucosan, we treated C2C12 cells with TM for 16 h and subsequently with or without α-amylase. Immunofluorescence staining revealed numerous ER stress-induced glycogen structures, which strongly colocalized with Stbd1 in control cells (Fig. 2A), whereas glycogen immunostaining was eliminated by α-amylase treatment (Fig. 2B). Nevertheless, despite the degradation of glycogen, structures exhibiting positive immunostaining for Stbd1, GS1 and GN were still detectable in α-amylase-treated cells (Fig. 2B). This suggests that glycogen contained on ER stress-induced clusters is sensitive to α-amylase treatment and therefore does not represent polyglucosan. Moreover, the finding that GS1 and GN remained colocalized with Stbd1 after the α-amylase-mediated depletion of glycogen may indicate that, during ER stress, the above proteins form a complex with Stbd1 at the ER membrane through direct protein–protein interactions that are not mediated by glycogen.

### Stbd1 is upregulated in response to ER stress and is required for the formation of glycogen structures

We have previously reported that transient overexpression of Stbd1 results in the accumulation of glycogen in organized smooth ER structures (Demetriadou et al., 2017). Given that ER stress induction in mouse myoblasts gives rise to comparable glycogen-containing structures, we hypothesized that ER stress activation may simulate a state of Stbd1 overexpression.

**Fig. 3.**
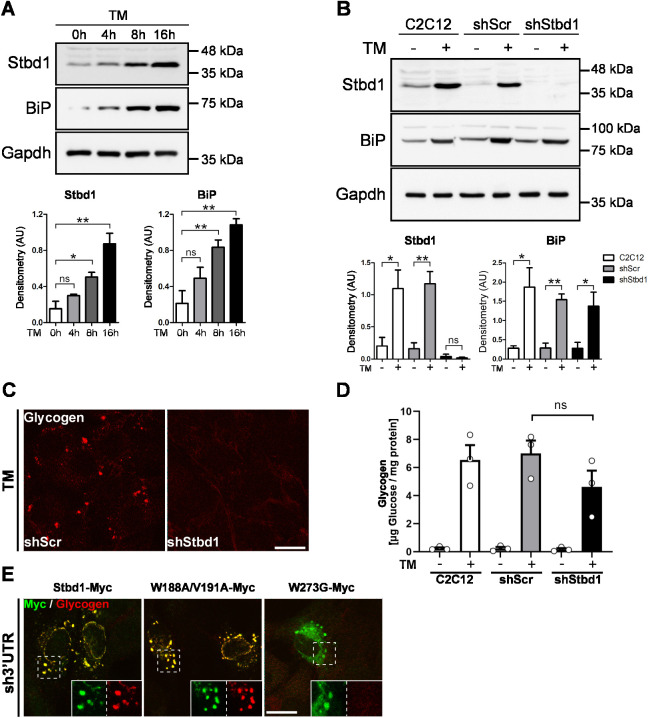
**Stbd1 is upregulated in response to ER stress and is required for the formation of glycogen structures.** (A) Western blot (top) and densitometry (bottom; mean±s.e.m., *n*=3) of protein extracts from C2C12 myoblasts collected after 0, 4, 8 or 16 h of TM treatment, showing prominent upregulation of Stbd1. Induction of ER stress was verified by the increase in BiP protein levels, and Gapdh was used as a loading control. (B) Western immunoblot (top) and densitometry (bottom; mean±s.e.m., *n*=3) for the assessment of Stbd1 silencing efficiency. Protein extracts from non-transduced controls (C2C12) and C2C12 myoblasts expressing either a scrambled (shScr) or a Stbd1-specific (shStbd1) shRNA sequence were prepared in the absence (−) or presence of TM treatment (+) and probed for Stbd1. Non-transduced and shScr cells display increased Stbd1 protein levels following ER stress activation, in contrast to shStbd1 cells. BiP was employed to evaluate ER stress activation, and Gapdh was used as a loading control. (C) Representative images of shScr and shStbd1 cells immunostained for glycogen after TM treatment. Stbd1 silencing impairs the formation of ER stress-induced glycogen clusters. (D) Quantification of glycogen levels in controls (non-transduced and shScr) and shStbd1 cells after treatment with DMSO (−) or TM (+), revealed no statistically significant difference (mean±s.e.m. μg glucose/mg protein: DMSO C2C12, 0.24±0.07; TM C2C12, 6.55±1.05; DMSO shScr, 0.23±0.11; TM shScr, 7.01±0.92; DMSO shStbd1, 0.17±0.08; TM shStbd1, 4.62±1.16; *n*=3). (E) Representative images of sh3′UTR C2C12 cells transiently transfected with the indicated vectors and immunostained for Myc and glycogen. Glycogen clustering is restored in cells transiently overexpressing Stbd1–Myc [mean±s.e.m. Manders’ colocalization coefficient (MCC): 0.800±0.026, *n*=10] or the W188A/V191A–Myc AIM mutant (MCC: 0.716±0.033, *n*=10) but not W273G–Myc (MCC: 0.051±0.014, *n*=10). Inserts show single stainings of the corresponding boxed areas at higher magnification. *P≤0.05; **P≤0.01; ns, not significant (one-tailed unpaired Student's *t*-test). Scale bars: 20 μm.

To address the above hypothesis, we treated C2C12 myoblasts with TM and evaluated Stbd1 protein levels at different time points, by means of western blotting. We found that Stbd1 displays low expression levels prior to ER stress induction but becomes gradually and highly upregulated during the course of TM treatment (Fig. 3A). Activation of the UPR pathway was confirmed by evaluating the protein levels of the early UPR chaperone BiP, which displayed a similar increase with time after TM addition (Fig. 3A).

Given that Stbd1 is an ER-resident transmembrane protein and that the ER is implicated in the formation of glycogen-containing structures in response to TM treatment, the ER stress-induced upregulation of Stbd1 could be the initiating step triggering the build-up of glycogen clusters. We therefore addressed the question of whether the above molecular events are Stbd1-dependent. To this end, we generated C2C12 myoblasts with stable knockdown of Stbd1 using a lentiviral shRNA approach. C2C12 cells expressing a scrambled shRNA sequence (shScr) were generated in parallel and used as controls. The silencing efficiency was evaluated by assessing Stbd1 upregulation in response to TM treatment. In contrast to non-transduced and shScr C2C12 myoblasts, which exhibited a prominent increase in Stbd1 protein levels, Stbd1-knockdown cells (shStbd1) did not display any obvious ER stress-induced Stbd1 upregulation, demonstrating efficient silencing (Fig. 3B). We next treated shStbd1 and shScr control myoblasts with TM and evaluated the formation of glycogen structures. Following ER stress induction by TM, shScr control cells displayed the typical intracellular glycogen clusters (Fig. 3C). In contrast, such structures were undetectable in shStbd1 myoblasts, suggesting that Stbd1 silencing compromises their build-up (Fig. 3C). Comparable results were obtained using TG as an ER stress inducer (Fig. S3A).

The failure to induce glycogen clustering upon ER stress activation in shStbd1 myoblasts raises the question of whether Stbd1 is required for ER stress-induced glycogen synthesis. To address this question, we quantified intracellular glycogen levels in non-transduced, shScr and shStbd1 C2C12 myoblasts treated with either TM or DMSO, as control. We found that, similar to non-transduced and shScr cells, shStbd1 cells displayed substantially increased glycogen content following TM treatment, as compared to the corresponding DMSO-treated controls (Fig. 3D). Glycogen levels in TM-treated Stbd1-knockdown cells appeared reduced when compared to similarly treated shScr myoblasts; however, the decrease was not statistically significant (Fig. 3D). The above data indicate that the absence of glycogen structures in TM-treated shStbd1 cells is not due to impaired glycogen synthesis and suggest a specific role for Stbd1 in glycogen clustering in response to ER stress.

To corroborate the above findings we performed rescue experiments using either wild-type (WT) or mutant Stbd1 variants. We employed the previously reported W273G glycogen-binding-deficient mutant (Jiang et al., 2010; Demetriadou et al., 2017) and a variant (W188A/V191A) carrying mutations in two conserved residues within the AIM motif, previously shown to abolish the interaction between human Stbd1 and Gabarapl1 (Jiang et al., 2011). Given that the target sequence initially used for Stbd1 silencing was within the coding region of the gene, the aforementioned shStbd1 cells could not be employed for rescue experiments. We therefore generated a second Stbd1-knockdown C2C12 cell line using a shRNA target sequence within the 3′ UTR (sh3′UTR). Efficient silencing in sh3′UTR myoblasts was confirmed by the lack of Stbd1 upregulation and glycogen clustering in response to TM treatment (Fig. S4A,B). Transient transfection of sh3′UTR cells with WT Stbd1 fused to a Myc epitope (Stbd1–Myc) restored glycogen clustering in 77.8% of transfected cells in the absence of ER stress activation by TM (Fig. 3E; Table S1). The number of transfected cells exhibiting glycogen structures increased to 92.1% following TM treatment (Table S1). The above is in line with the low levels of glycogen in C2C12 cells under basal conditions, which become substantially increased upon ER stress activation. A similar degree of rescue (73% and 87.9% in the absence and presence of TM, respectively) was observed with W188A/V191A–Myc (Fig. 3E; Table S1). However, no obvious glycogen clustering was evident with the W273G–Myc glycogen-binding mutant, either in the absence or presence of TM (Fig. 3E; Table S1). This indicates that, although the glycogen-binding domain of Stbd1 is required for the formation of glycogen structures, a functional AIM motif is not essential.

### Inhibition of PERK signalling compromises ER stress-induced Stbd1 upregulation and glycogen clustering

**Fig. 4.**
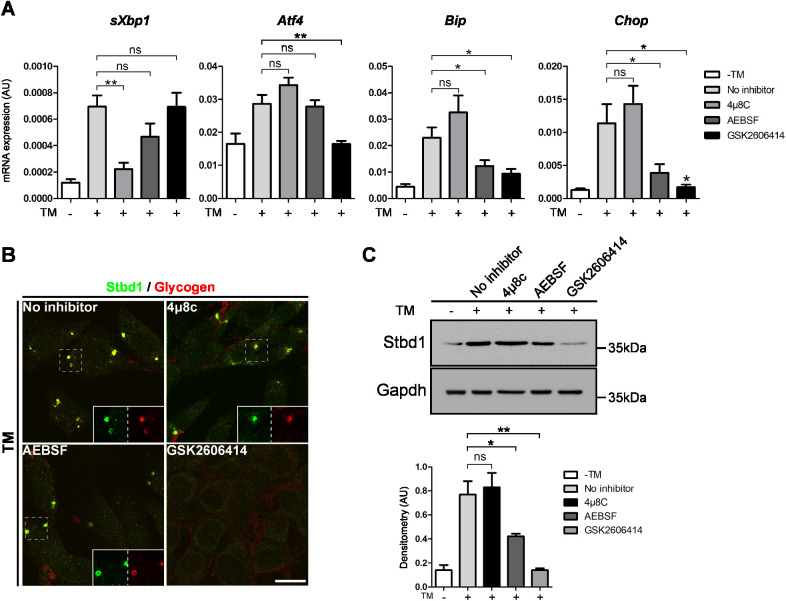
**ER stress-induced Stbd1 upregulation and glycogen clustering depends on PERK signalling.** C2C12 cells were either left untreated (−) or cultured with TM (+) in the absence or presence of inhibitors of the individual UPR branches. (A) mRNA expression levels of representative UPR marker genes as assessed by qPCR. Data represent mean±s.e.m. of three independent experiments each performed in triplicate. (B) Representative images of double immunostaining for Stbd1 and glycogen revealed the lack of ER stress-induced, Stbd1-positive glycogen structures in the presence of the PERK inhibitor GSK2606414 [mean±s.e.m. Manders’ colocalization coefficient (MCC): no inhibitor, 0.870±0.011, *n*=10; 4μ8C, 0.768±0.021, *n*=10; AEBSF, 0.721±0.019, *n*=10]. Single stainings are shown as inserts representing higher magnifications of the corresponding boxed areas. (C) Western blot (top) and densitometry (bottom; mean±s.e.m., *n*=3) of protein extracts from cells treated as described above confirms the lack of ER stress-induced Stbd1 upregulation in the presence of the selective PERK inhibitor and a partial inhibition with AEBSF. Gapdh is shown as a loading control. *P≤0.05; **P≤0.01; ns, not significant (one-tailed unpaired Student's *t*-test). Scale bar: 20 μm.

We next sought to identify the UPR signalling branch through which Stbd1 upregulation is mediated using inhibitors of the individual pathways. For the inhibition of IRE1, ATF6 or PERK signalling we employed 4μ8C (Cross et al., 2012), AEBSF (Okada et al., 2003) or GSK2606414 (Axten et al., 2012), respectively. The efficacy of the above inhibitors was addressed by assessing the mRNA expression levels of representative ER stress response genes [*sXbp1* (the spliced form of *Xbp1*), *Atf4*, *BiP* and *Chop* (also known as *Ddit3*)] by means of qPCR. *sXbp1* is a key marker of IRE1 activation (Calfon et al., 2002), *Atf4* is a downstream effector of PERK (Harding et al., 2000), whereas *BiP* is a known target of the ATF6 signalling branch (Wu et al., 2007; Yamamoto et al., 2007). The pro-apoptotic factor *Chop* is a further downstream target of the PERK pathway that is directly activated by ATF4 and also by ATF6 (Yoshida et al., 2000; Han et al., 2013). As expected, the IRE1 inhibitor 4μ8C specifically blocked the TM-induced activation of *sXbp1*, whereas AEBSF attenuated the induction of both *BiP* and *Chop* (Fig. 4A). GSK2606414 specifically compromised the ER stress-mediated activation of the PERK targets *Atf4* and *Chop* and further inhibited the expression of *BiP* (Fig. 4A). A similar partial dependence of ER stress-mediated upregulation of *BiP* on PERK signalling has been demonstrated previously in mouse embryonic fibroblasts (Gonen et al., 2019).

To identify the UPR pathway promoting Stbd1 upregulation we exploited the absolute requirement for Stbd1 for the ER stress-induced formation of glycogen structures. C2C12 myoblasts were therefore treated with TM in the presence of the above inhibitors and evaluated for the formation of Stbd1-positive glycogen clusters. Inhibition of either IRE1 or ATF6 signalling did not impinge on the build-up of Stbd1-positive glycogen clusters following TM treatment (Fig. 4B). Nevertheless, in the presence of the selective PERK inhibitor GSK2606414, glycogen structures failed to form, suggesting that activation of Stbd1 expression upon ER stress induction depends on PERK signalling (Fig. 4B). A similar result was obtained with TG treatment (Fig. S3B). The above findings were corroborated by western blotting, which revealed that the TM-induced Stbd1 upregulation was not inhibited by 4μ8C but was completely abolished upon treatment with GSK2606414 (Fig. 4C). A milder but statistically significant attenuation of Stbd1 upregulation was observed in the presence of AEBSF (Fig. 4C). Nevertheless, this partial ER stress-mediated activation of Stbd1 expression upon inhibition of ATF6 signalling was apparently sufficient to induce glycogen clustering (Fig. 4B).

### Stbd1 overexpression in C2C12 myoblasts is sufficient to induce glycogen clustering independently of ER stress activation

The results described above demonstrate that Stbd1 is required for ER stress-induced glycogen clustering in C2C12 myoblasts. However, Stbd1 silencing does not appear to abolish glycogenesis, suggesting that glycogen synthesis and clustering in response to ER stress occur independently.

**Fig. 5.**
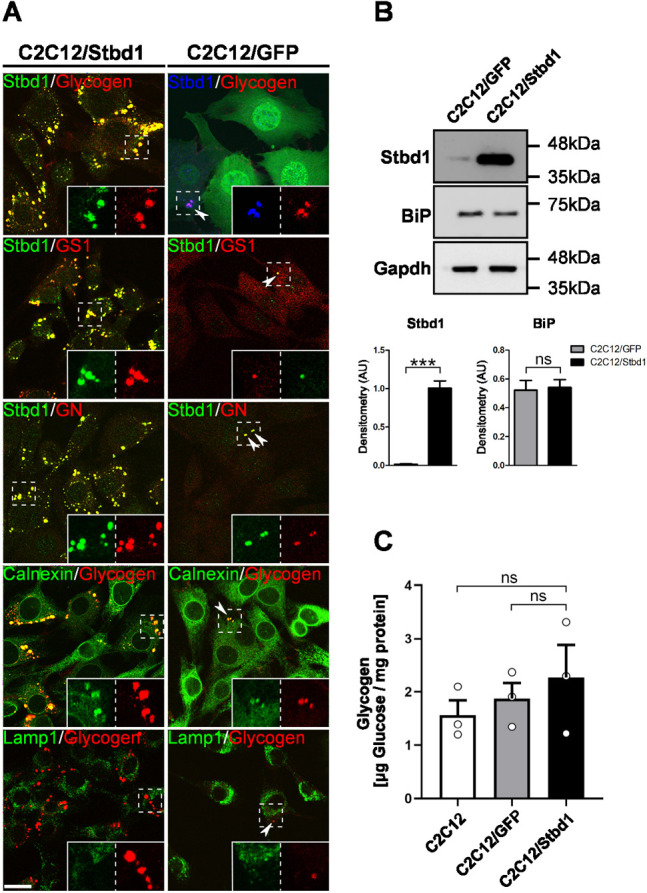
**Stbd1 overexpression is sufficient to induce glycogen clustering in the absence of ER stress but does not stimulate glycogenesis.** (A) Representative images of C2C12 myoblasts stably overexpressing mouse Stbd1 (C2C12/Stbd1) or GFP as control (C2C12/GFP), stained using the indicated antibodies. C2C12/Stbd1 cells display large glycogen clusters that resemble those induced by ER stress and stain positive for Stbd1 [mean±s.e.m. Manders’ colocalization coefficient (MCC): 0.796±0.014, *n*=10], GS1 (MCC: 0.792±0.019, *n*=10), GN (MCC: 0.745±0.014, *n*=10), calnexin (MCC: 0.774±0.013, *n*=10) but not Lamp1 (MCC: 0.089±0.005, *n*=10). C2C12/GFP controls sporadically display smaller structures (arrowheads), with similar immunofluorescence staining for the above markers (MCC: Stbd1, 0.570±0.044, *n*=10; GS1, 0.618±0.021, *n*=8; GN, 0.518±0.033, *n*=8; calnexin, 0.646±0.053, *n*=5; Lamp1, 0.035±0.015, *n*=7). Inserts show single stainings of the corresponding boxed areas at higher magnification. (B) Western blot (top) and densitometry (bottom; mean±s.e.m., *n*=3) of protein lysates from C2C12/GFP and C2C12/Stbd1 cells confirms Stbd1 overexpression and the absence of ER stress activation in C2C12/Stbd1 cells, as assessed by BiP protein levels. Gapdh is shown as a loading control. (C) Quantification of intracellular glycogen levels in non-transduced C2C12, C2C12/GFP and C2C12/Stbd1 cells. Glycogen content in Stbd1-overexpressing cells is not statistically different from that of controls (mean±s.e.m, μg glucose/mg protein: C2C12, 1.56±0.27; C2C12/GFP, 1.87±0.30; C2C12/Stbd1, 2.27±0.60; *n*=3). ***P≤0.001; ns, not significant (one-tailed unpaired Student's *t*-test). Scale bar: 20 μm.

To address the above, we generated stable C2C12 myoblast cells overexpressing either mouse Stbd1 (C2C12/Stbd1) or the unrelated GFP protein (C2C12/GFP) as control, by means of lentiviral infection. Immunofluorescence staining revealed the presence of numerous large glycogen structures in C2C12/Stbd1 cells, which stained positive for Stbd1, GS1, GN and calnexin, but not Lamp1, and thus highly resembled the Stbd1-dependent glycogen structures induced by ER stress (Fig. 5A). In contrast, C2C12/GFP controls did not contain comparable clusters but did occasionally display smaller glycogen structures with a similar immunofluorescence staining pattern (Fig. 5A, arrowheads).

Given that TM treatment and Stbd1 overexpression gave rise to similar glycogen structures, we evaluated whether Stbd1-overexpressing myoblasts display UPR activation. As revealed by western blotting, both C2C12/Stbd1 and C2C12/GFP control myoblasts displayed similar protein levels of the UPR marker BiP, suggesting that Stbd1 overexpression did not provoke UPR activation (Fig. 5B). As in the case of glycogen structures induced by ER stress, α-amylase treatment of C2C12/Stbd1 myoblasts resulted in the complete degradation of glycogen, whereas Stbd1, GS1 and GN remained colocalized on intracellular structures devoid of glycogen (Fig. S5). The above findings demonstrate that, in the absence of ER stress, Stbd1 is sufficient to induce the formation of ER structures containing α-amylase-degradable glycogen and the glycogenic proteins GN and GS1. Moreover, the presence of GS1 and GN on Stbd1-positive structures following the degradation of glycogen by α-amylase further supports that Stbd1 may form a scaffold at the ER membrane mediating the recruitment of GS1 and GN, likely through direct protein–protein interactions.

We next examined whether Stbd1 overexpression and the concomitant recruitment of GN and GS1 to the ER membrane is associated with an increase in intracellular glycogen content. For this, we quantified glycogen levels in C2C12/Stbd1 myoblasts, non-transduced C2C12 and C2C12/GFP control cells. We found that C2C12/Stbd1 cells did not display a significant increase in glycogen levels (Fig. 5C). This suggests that Stbd1 overexpression is sufficient to promote glycogen clustering and the recruitment of GN and GS1 to the ER membrane in the absence of ER stress, but additional ER stress-mediated processes are required to stimulate glycogenesis.

### Stbd1 is not required for PTG-induced glycogen accumulation

The herein described role for Stbd1 in promoting the formation of glycogen structures in response to ER stress activation raises the question of whether Stbd1 has a specific role during ER stress or is generally implicated in glycogen clustering.

A number of previous studies established an important role for the adaptor protein PTG in the regulation of glycogen levels in tissues. PTG targets protein phosphatase 1 (PP1) to glycogen, resulting in the dephosphorylation and reciprocal modulation of the enzymatic activities of glycogen synthase (activation) and phosphorylase (inhibition) causing a significant increase in glycogen levels (Printen et al., 1997; Lerin et al., 2000).

**Fig. 6.**
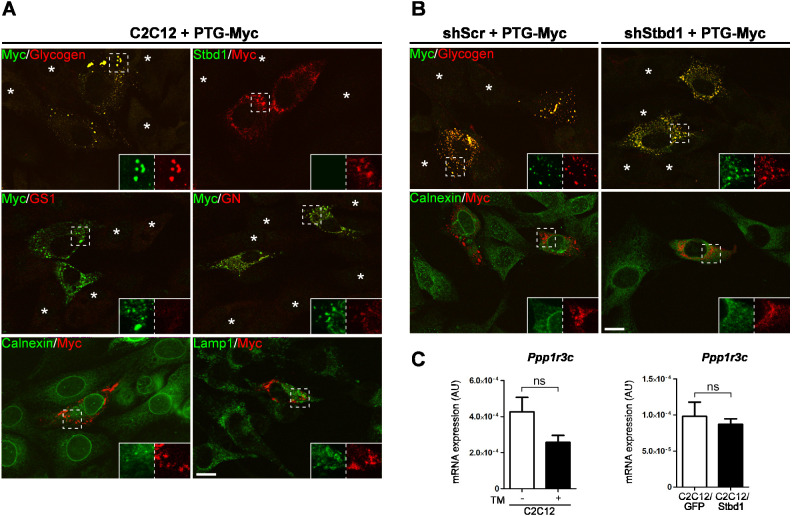
**Stbd1 is not required for PTG-induced glycogen accumulation.** (A) C2C12 myoblasts transiently transfected with PTG–Myc and immunostained with the indicated antibodies. PTG overexpression induces the build-up of glycogen clusters strongly colocalizing with PTG [mean±s.e.m. Manders’ colocalization coefficient (MCC): 0.775±0.023, *n*=10]. PTG-induced glycogen structures do not display any detectable Stbd1 (MCC: 0.024±0.006, *n*=10) or GS1 (MCC: 0.114±0.012, *n*=10) immunofluorescence; however, they stain weakly positive for GN (MCC: 0.624±0.013, *n*=10). No colocalization is observed with calnexin (MCC: 0.009±0.003, *n*=10) or Lamp1 (MCC: 0.067±0.010, *n*=10). (B) shScr and shStbd1 C2C12 cells transiently transfected with PTG–Myc and double stained for the indicated antibodies. PTG overexpression induces the formation of glycogen structures in the absence of Stbd1 (MCC: shScr, 0.658±0.022, *n*=10; shStbd1, 0.784±0.029, *n*=10) which do not coincide with calnexin (MCC: shScr, 0.014±0.002, *n*=10; shStbd1, 0.107±0.018, *n*=10). In A and B, representative images are shown. Asterisks indicate non-transfected cells. Inserts show single stainings of the corresponding boxed areas at higher magnification. (C) mRNA expression levels of *Ppp1r3c* in TM-treated (+) and Stbd1-overexpressing C2C12 myoblasts, as compared to controls [non-treated (−) and GFP-overexpressing cells, respectively]. Data represent mean±s.e.m. of four independent experiments each performed in triplicate. ns, not significant (one-tailed unpaired Student's *t*-test). Scale bars: 20 μm.

To explore the potential implication of Stbd1 in the formation of PTG-induced glycogen clusters, we constructed an expression vector in which the coding sequence of PTG was fused to a Myc epitope at its C-terminus (PTG–Myc). As expected, overexpression of PTG–Myc resulted in massive accumulation of glycogen clusters in C2C12 cells (Fig. 6A). These strongly coincided with PTG–Myc; however, they stained negative for both Stbd1 and GS1 (Fig. 6A). This suggests that PTG likely promotes the formation of glycogen clusters independently of Stbd1 and further supports that recruitment of GS1 to glycogen structures is likely mediated by Stbd1. Nevertheless, glycogen structures in PTG–Myc-transfected cells were weakly positive for GN, probably reflecting the fact that GN is an integrated component of glycogen. Furthermore, PTG-induced glycogen structures displayed no colocalization with calnexin, suggesting that they are not associated with the ER, and no staining for Lamp1, similar to the clusters induced by ER stress (Fig. 6A).

We next addressed a potential role for Stbd1 in the build-up of PTG-induced glycogen structures by transiently overexpressing PTG–Myc in shStbd1 and shScr control myoblasts. PTG–Myc overexpression in shStbd1 myoblasts induced the accumulation of glycogen, as in shScr cells, which demonstrates that Stbd1 is not required for the formation of PTG-induced glycogen clusters (Fig. 6B). Moreover, similar to shScr controls, accumulated glycogen clusters in shStbd1 myoblasts did not colocalize with calnexin (Fig. 6B). The above data suggest that Stbd1 is not generally implicated in glycogen clustering but, rather, has a specific role in the formation of glycogen structures induced by ER stress. To rule out potential secondary effects of ER stress activation and Stbd1 overexpression on PTG, we assessed the expression levels of the PTG-encoding gene *Ppp1r3c* in TM-treated and Stbd1-overexpressing cells using qPCR. This revealed that neither TM treatment nor Stbd1 overexpression significantly affected *Ppp1r3c* expression as compared to *Ppp1r3c* levels in non-treated and GFP-overexpressing C2C12 myoblasts, respectively (Fig. 6C).

### Deficiency in the formation of ER stress-induced, Stbd1-dependent glycogen structures is associated with enhanced susceptibility to apoptosis

**Fig. 7.**
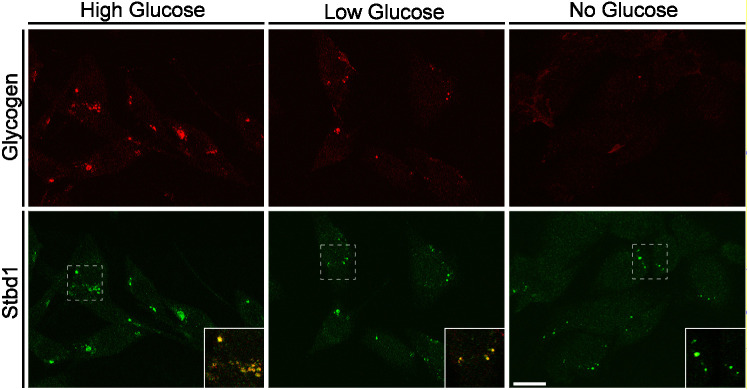
**ER stress-induced glycogen structures are degraded under conditions of glucose restriction.** C2C12 myoblasts were treated with TM for 16 h and cultured for an additional 6 h in medium containing either high (25 mM), low (5 mM) or no glucose, in the absence of TM. Representative images of double immunofluorescence staining for glycogen and Stbd1 are shown. Compared to the glycogen clusters in cells cultured in high-glucose medium, ER stress-induced structures appear smaller under low-glucose conditions and were almost completely devoid of glycogen in the absence of glucose. Despite the near-complete degradation of glycogen under glucose-free conditions, structures induced by ER stress displayed positive immunostaining for Stbd1 [mean±s.e.m. Manders’ colocalization coefficient (MCC): high glucose, 0.764±0.018, *n*=10; low glucose, 0.772±0.013, *n*=10; no glucose, 0.202±0.045, *n*=10]. Overlays are shown as inserts and represent higher magnification of the corresponding boxed areas. Scale bar: 20 μm.

Our results demonstrate that in mouse myoblasts, ER stress triggers the synthesis of glycogen and its Stbd1-dependent accumulation on ER membranes. To address the biological significance of this cellular response to ER stress, we first examined whether and under which conditions the ER stress-induced glycogen structures resolve. For this, we subjected C2C12 myoblasts to TM treatment for 16 h, to induce the formation of glycogen clusters, and further cultured the cells for an additional 6 h in the presence of either high (25 mM), low (5 mM) or no glucose. Myoblasts cultured in high glucose after the removal of TM still displayed large intracellular glycogen structures (Fig. 7). However, in cells incubated in low glucose, glycogen clusters appeared smaller and they were almost completely resolved in the absence of glucose (Fig. 7). Importantly, despite the degradation of glycogen, glucose-deprived cells still displayed Stbd1-positive puncta (Fig. 7). The above findings indicate that glycogen present on ER stress-induced structures is degraded under conditions of glucose restriction following the removal of the ER stressor. Comparable results were obtained with TG treatment (Fig. S3C).

**Fig. 8.**
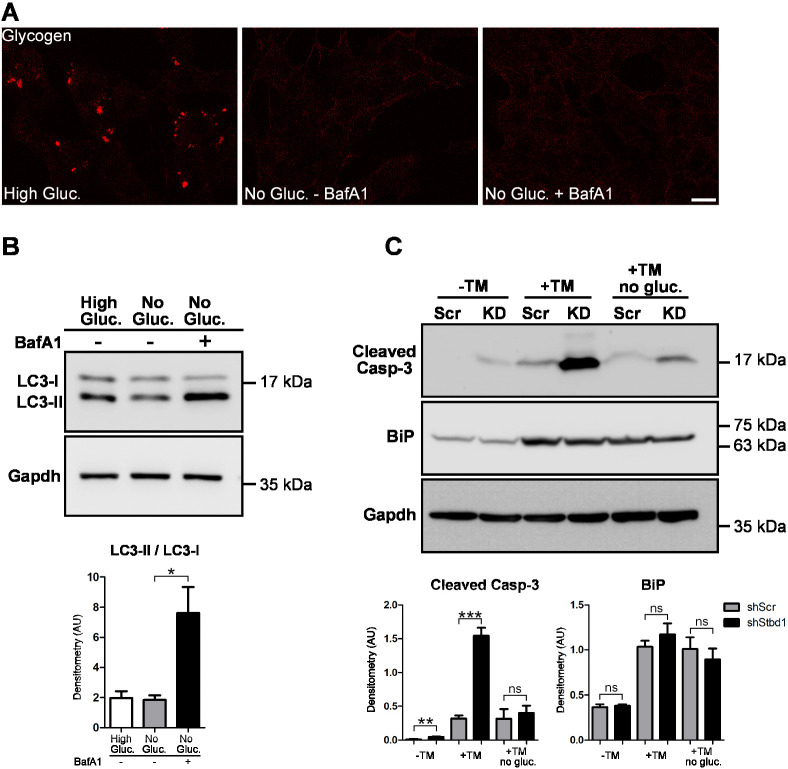
**Formation of glycogen-containing clusters** **is associated with enhanced cell survival during ER stress.** (A) Representative immunofluorescence staining for glycogen performed on C2C12 myoblasts treated with TM for 16 h followed by TM withdrawal and additional culturing for 6 h in either medium containing 25 mM glucose (high gluc.) or glucose-free medium in the absence (no gluc.−BafA1) or presence (no gluc.+BafA1) of BafA1. ER stress-induced glycogen is resolved in the presence of BafA1. (B) Western blot (top) and densitometry (bottom; mean±s.e.m., *n*=3) of lysates from C2C12 cells treated as described above and probed for LC3 revealed elevated LC3-II/LC3-I ratio in the presence of BafA1, thus confirming inhibition of autophagic flux. Gapdh is shown as a loading control. (C) Western blot (top) and densitometry (bottom; mean±s.e.m., *n*=3) of protein lysates from shScr and shStbd1 (KD) C2C12 cells at basal conditions (−TM), after 16 h of TM treatment (+TM) or after 16 h of TM treatment followed by TM withdrawal and culturing for 6 h in glucose-free medium (+TM no gluc.) reveals significantly elevated cleaved Casp-3 protein levels in shStbd1 cells, particularly after TM treatment. ER stress activation by TM was confirmed by monitoring the protein levels of BiP. Gapdh is shown as a loading control. *P≤0.05; **P≤0.01; ***P≤0.001; ns, not significant (one-tailed unpaired Student's *t*-test). Scale bar: 20 μm.

Based on the presence of an AIM motif, Stbd1 has been proposed to act as a selective autophagy receptor for glycogen, mediating its targeting to lysosomes (Jiang et al., 2011). Given the above, we evaluated whether glycogen structures induced by ER stress in C2C12 myoblasts resolve in the presence of Bafilomycin A1 (BafA1), an autophagy inhibitor that prevents lysosomal acidification and interferes with the fusion between autophagosomes and lysosomes (Yamamoto et al., 1998). Treatment of C2C12 cells with TM promoted the formation of the typical intracytoplasmic glycogen clusters, which remained detectable following TM withdrawal and culturing of the cells in high-glucose-containing medium (Fig. 8A). These structures completely resolved within 6 h of incubation without glucose both in the absence and presence of BafA1, suggesting that their degradation does not depend on an autophagic process entailing autophagosome–lysosome fusion (Fig. 8A). Inhibition of autophagy by BafA1 was verified by western blotting, which revealed significantly increased levels of lipidated LC3 (LC3-II), indicating autophagosome accumulation due to impaired autophagic flux (Fig. 8B).

Given that ER stress activation induces the formation of glycogen structures, which resolve under conditions of glucose starvation, we addressed the question of whether their formation and/or availability for enzymatic degradation impinge on cell survival. For this, we assessed the activation of the apoptotic cell death program in shStbd1 myoblasts, in which ER stress-induced glycogen clusters fail to form, as compared to shScr control cells. We evaluated the levels of the apoptotic executor activated/cleaved caspase 3 (Casp-3) at basal conditions, after 16 h of TM treatment and 6 h after TM withdrawal and culturing in glucose-free medium. Under basal conditions, cleaved Casp-3 levels were already higher in shStbd1 cells, suggesting increased basal apoptosis (Fig. 8C). Although activation of the UPR pathway primarily aims to restore ER homeostasis, it is well established that it can also induce cell death (Iurlaro and Muñoz-Pinedo, 2016). Accordingly, TM treatment resulted in increased cleaved Casp-3 levels in shScr controls, as compared to basal levels, whereas a significantly more prominent Casp-3 activation was evident in TM-treated shStbd1 myoblasts (Fig. 8C). TM withdrawal and cultivation under glucose-free conditions ameliorated activation of apoptotic cell death, as assessed by cleaved Casp-3 levels in both shScr and shStbd1 cells (Fig. 8C). Nevertheless, shStbd1 cells still displayed increased apoptotic signalling, as compared to shScr controls (Fig. 8C). Similar findings were obtained with TG treatment (Fig. S3D).

The above results suggest that Stbd1-silenced myoblasts are more susceptible to apoptotic cell death compared to shScr controls. This becomes particularly pronounced during the course of ER stress and is not further enhanced but instead mitigated following withdrawal of the ER stressor and culturing of cells in glucose-free medium. Of note, the above two conditions are associated with the formation and resolution of ER stress-induced glycogen structures, respectively. This may indicate an important correlation between the build-up of glycogen-containing clusters and support of cell survival during ER stress.

## DISCUSSION

The UPR pathway is a cellular adaptation mechanism that primarily aims to promote cell survival under conditions of ER stress. Genes encoding enzymes involved in glucose and glycogen metabolism have been previously recognized among UPR targets (Acosta-Alvear et al., 2007; Lee et al., 2008). However, the relationships between ER stress and glycogen metabolism are poorly understood. As previously reported, ER stress activation in N2A neuroblastoma cells induces glycogenesis and the build-up of glycogen structures consisting of polyglucosan (Wang et al., 2013). In the present study, we show that ER stress activation in mouse myoblasts elicits a similar response, that is, a significant increase in cellular glycogen levels and formation of glycogen-positive structures. This suggests that glycogen synthesis and clustering in response to UPR activation occurs in different cell types and lineages. However, cell-type specific differences seem to exist with regards to the structure of the synthesized glycogen. Whereas glycogen formed in N2A cells was reported to be α-amylase-resistant (Wang et al., 2013), our findings demonstrate that ER stress-induced structures in C2C12 myoblasts harbour α-amylase-degradable glycogen. Although the basis of the above discrepancy is not clear at this stage, it could be related to cell-type-specific differences in the activity or regulation of enzymes promoting glycogen synthesis and branching.

Data presented in this study reveal a new, specific role for Stbd1 during the ER stress response of mouse myoblasts in the formation of glycogen-containing structures. This is exemplified by the finding that Stbd1 silencing compromised glycogen clustering but did not significantly influence the TM-stimulated increase in intracellular glycogen levels. Moreover, in the absence of UPR activation, Stbd1 was sufficient to promote the formation of similar glycogen clusters when overexpressed in C2C12 cells. Importantly, Stbd1 overexpression did not lead to increased glycogen content. This suggests that formation of glycogen-containing structures in Stbd1-overexpressing myoblasts occurs independently of glycogen synthesis stimulation, likely through the Stbd1-mediated recruitment of existing intracellular glycogen to the ER membrane. The above findings further indicate that, although Stbd1 is both necessary and sufficient to promote glycogen clustering, additional UPR-mediated processes are required to stimulate glycogenesis during ER stress. It has been previously reported that induction of ER stress in N2A cells results in a major increase in the activity of GS1 (Wang et al., 2013). The absence of UPR activation could thus explain the lack of markedly elevated glycogen levels in Stbd1-overexpressing myoblasts and implies that GS1 requires additional activation through ER stress-dependent processes.

Our results indicate that glycogen structures formed both in response to TM treatment and stable Stbd1 overexpression stain positive for Stbd1, the glycogenic proteins GN and GS1 and the ER marker calnexin. The formation of these glycogen-containing clusters was also induced by other ER stressors, such as 2-DG and TG, suggesting that it comprises a general response to ER stress activation that does not depend on the molecular mechanism perturbing ER homeostasis. Nevertheless, the type of ER stressor and mechanism of UPR activation (TM and 2-DG interfere with protein glycosylation, whereas TG results in the depletion of ER calcium) appear to influence the immunostaining pattern of calnexin. While glycogen-containing structures induced by TM and 2-DG are uniformly stained with calnexin, ER stress activation by TG results in both calnexin-positive and calnexin-negative structures. The basis of the above inconsistency is currently elusive. However, it could be related to different effects of the individual ER stressors on ER morphology, extent and intensity of ER stress or the subcellular distribution of calnexin. Differential effects of TM and TG on ER morphology were previously reported in a study that revealed the occurrence of transient ER structures during C2C12 myoblast differentiation. These structures, which were termed SARC (stress-activated response to Ca^2+^ depletion) bodies were shown to occur upon ER stress activation specifically by TG but not TM (Nakanishi et al., 2015). SARC bodies display no immunostaining for calnexin or other typical ER markers (Nakanishi et al., 2015). The above is in agreement with our observation that calnexin-negative structures form following ER stress induction by TG but not TM or 2-DG.

The fact that glycogen structures induced either by ER stress or Stbd1 overexpression in C2C12 myoblasts are strikingly similar suggests that activation of ER stress results in a state of Stbd1 overexpression. Indeed, we provide evidence that Stbd1 is markedly upregulated following ER stress induction and that its upregulation depends on PERK signalling. Our results further demonstrate that following glycogen depletion by α-amylase, ER structures induced by either TM or Stbd1 overexpression still display positive immunostaining for Stbd1, GN and GS1, suggesting that Stbd1 might serve as a scaffold mediating the recruitment of GS1 and GN to the ER membrane through direct protein–protein interactions. Interactions between human Stbd1 and a number of glycogen-binding proteins such as GS1, glycogen debranching enzyme and laforin have been previously reported (Zhu et al., 2014). Moreover, a high-throughput mass spectrometry approach confirmed the above interactions and identified additional Stbd1-binding partners, which include GN-1 (GYG1) and GN-2 (GYG2) (Huttlin et al., 2017). It is therefore likely that GS1 and GN are components of a larger macromolecular complex that assembles at the ER membrane in a Stbd1-dependent manner in response to UPR activation or Stbd1 overexpression and that might also consist of additional Stbd1-interacting proteins. Based on the above findings, the build-up of glycogen structures in response to ER stress could be compatible with a process initiated by the PERK-dependent upregulation of Stbd1. This would result in a spatial accumulation of Stbd1 at the ER membrane that generates platforms for glycogen clustering and the assembly of a protein complex encompassing GN and GS1. As shown in Stbd1-overexpressing myoblasts, Stbd1 is sufficient to recruit existing cellular glycogen to the ER. Alternatively, or in addition to the above, the ER stress-induced, Stbd1-dependent targeting of GN and GS1 to the ER may initiate the assembly of a protein complex promoting glycogen synthesis at the ER membrane contributing to the total cellular glycogen pool. Either scenario (recruitment of cytosolic glycogen or glycogen synthesis at the ER membrane) would be compatible with our finding that Stbd1 silencing does not significantly influence ER stress-induced glycogen levels, because in both cases GS1 could still promote glycogen synthesis in the cytosol.

Data reported in this study suggest that Stbd1 is specifically required for the formation of glycogen structures during ER stress. This is supported by the findings that glycogen clusters generated by the overexpression of PTG occurred in the absence of Stbd1. Moreover, PTG-induced glycogen clusters did not appear to coincide with either Stbd1 or calnexin, indicating that these are not associated with the ER. It has been recognized very early on that glycogen is not evenly distributed within cells but displays compartmentalization. A number of studies reported that in addition to ‘free’ cytosolic glycogen, a fraction of glycogen granules displays close association with ER membranes in both liver and muscle cells (Fawcett, 1955; Wanson and Drochmans, 1972; Margolis et al., 1979; Goldstein et al., 1985). Furthermore, the fraction of glycogen associated with the sarcoplasmic reticulum (SR) was shown to be elevated in skeletal muscle of streptozotocin-injected diabetic rats, suggesting that localization of glycogen to the SR is influenced by the metabolic status of the organism (Garduño et al., 2001). It is currently not known how the association of glycogen with the ER is mediated. A compelling speculation is that this attachment occurs through its binding to an ER-associated protein, such as Stbd1.

Our data raise the question concerning the biological significance of ER stress-induced synthesis of glycogen and its Stbd1-dependent clustering in myoblasts. We showed that Stbd1-silenced myoblasts, in which the build-up of glycogen-containing structures was compromised, displayed increased levels of activated/cleaved Casp-3 following ER stress induction. This suggests that Stbd1 knockdown renders cells susceptible to apoptotic cell death, particularly under conditions of ER stress, and that glycogen clustering may become important for cell survival. Data reported in this study indicate that ER stress-induced glycogen structures are degraded under conditions of glucose restriction, suggesting that they can serve as glucose reserves. However, despite the lack of these structures in Stbd1-knockdown cells, apoptotic signalling under glucose-free conditions is not further exacerbated but instead improved. This suggests that glycogen clusters generated during ER stress do not considerably contribute to support glucose demands under glucose-free conditions. As shown in this study, ER stress activation in C2C12 myoblasts results in a substantial increase in cellular glycogen levels that is not significantly affected by Stbd1 silencing. This suggests that, despite the absence of ER stress-induced glycogen structures, Stbd1-silenced cells harbour ample amounts of cytosolic glycogen generated during ER stress, which could explain their recovery from apoptotic stimuli under glucose-free conditions.

ER stress results in the activation of several pro-survival pathways, which are associated with elevated energy demands such as the increased synthesis and folding of newly expressed ER chaperones and downstream UPR targets. As previously shown, ER stress induction by TM results in the tightening of ER–mitochondria contacts and the relocation of mitochondria to perinuclear ER regions (Bravo et al., 2011). These redistributed mitochondria were reported to exhibit increased ATP production to meet high, regional requirements of the stressed ER for ATP. In addition to energy, protein glycosylation is central to the proper folding and maturation of proteins within the ER and is of pivotal importance for ER homeostasis. Similar to the increase in ER–mitochondria contacts during ER stress, ER stress-induced glycogen-containing structures could support the regional and direct provision of glucose to cope with the increased glucose demand for glycosylation reactions in the ER.

We have previously demonstrated that, in addition to being targeted to bulk ER, Stbd1 also localizes to ER–mitochondrial contact sites (Demetriadou et al., 2017). A number of studies support a direct link between changes in the composition of ER–mitochondria contact sites or ER–mitochondria association and sensitivity to apoptosis during ER stress (Chami et al., 2008; Sano et al., 2009; Calì et al., 2013). Thus, alternatively or in addition to its role in promoting glycogen clustering at the ER, Stbd1 might impact on cell viability during ER stress by influencing the physical contacts between ER and mitochondria and/or processes related to ER–mitochondrial communication.

For myoblasts, glycogen synthesis in response to ER stress may be of particular importance. Myoblasts are undifferentiated cells that have the capacity to withdraw from the cell cycle, fuse with each other and differentiate into myotubes. The above process is important for both skeletal muscle development during embryogenesis and regeneration following muscle injury. ER stress was shown to be transiently activated during normal myoblast differentiation and required to induce apoptosis to selectively eliminate differentiation-incompetent myoblasts (Nakanishi et al., 2005, 2007). Furthermore, proper myogenic differentiation depends on glucose availability, because it is inhibited by glucose restriction (Fulco et al., 2008; Nedachi et al., 2008). ER stress-induced glycogen synthesis and accumulation could thus be an integral mechanism of myogenesis, ensuring the availability of intracellular glucose supply to support myoblast survival and differentiation.

Although experimental evidence is lacking, based on the presence of an AIM motif and its ability to bind glycogen, Stbd1 is thought to function as a selective autophagy receptor for glycogen, in a process termed glycophagy (Jiang et al., 2011). The above is also supported by the localization of Stbd1 to ER–mitochondria contact sites, which have been identified as the sites at which autophagosomes originate (Hamasaki et al., 2013). Thus, the involvement of the ER membrane in the build-up of glycogen-containing structures in response to ER stress could promote the accumulation of glycogen at ER sites, resulting in its inclusion into autophagosomes for lysosomal degradation. However, our results indicate that ER stress-induced glycogen structures do not colocalize with Lamp1. Moreover, their clearance under conditions of glucose deprivation does not depend on autophagosome–lysosome fusion, suggesting that they are most likely not degraded by the classical macroautophagy pathway.

ER stress activates complex, multifaceted signalling networks that aim to restore cellular homeostasis. We have identified the formation of Stbd1-dependent glycogen structures at the ER membrane as a component of the cellular response of mouse myoblasts to ER stress and have shown that Stbd1 contributes to cell survival. Although mechanistic insights are currently lacking, this process could support ER homeostatic functions and/or cellular metabolic requirements.

## MATERIALS AND METHODS

### Cell lines

C2C12 myoblasts and HEK293T cells were obtained from the American Type Culture Collection (ATCC) and cultured in high glucose (25 mM) Dulbecco's modified Eagle's medium (DMEM; Biosera) supplemented with 10% fetal bovine serum (FBS; Biosera), 1×penicillin-streptomycin and 1×glutamine (Biosera) in a humidified chamber at 37°C under 5% CO_2_. Cell lines were tested for mycoplasma (PCR Mycoplasma test kit I/C, Promokine) and were found to be free of contamination.

### Plasmid construction

For the generation of an expression vector for *PTG* (*Ppp1r3c*, NM_016854), the corresponding coding region was amplified by PCR from mouse liver cDNA and inserted into pcDNA3.1/myc-HisA (Invitrogen), in frame with a C-terminal Myc epitope. The AIM mutant variant (W188A/V191A–Myc) was generated by site-directed mutagenesis from WT Stbd1–Myc (Demetriadou et al., 2017) using a Q5 site-directed mutagenesis kit (New England Biolabs) according to the instructions of the manufacturer. A lentiviral vector for the expression of *Stbd1* under the control of the human *PGK1* promoter was constructed by replacing *GFP* in the pCCLsin.cPPT.hPGK.eGFP.Wpre vector (Santoni de Sio et al., 2006) with the coding region of mouse *Stbd1*.

### Antibodies

The following primary antibodies were used in this study: anti-Stbd1 (11842-1-AP; Proteintech; 1:500), anti-calnexin (ADI-SPA-860-D; Enzo Life Sciences; 1:250), anti-Lamp1 (1D4B; Developmental Studies Hybridoma Bank; 1:50) and anti-LC3 (NB100-2220; Novus Biologicals; 1:435). Antibodies specific for GS1 (GS-7H5, SC-91173; 1:200), glycogenin-1 (E-11, sc-271109; 1:200), Gapdh (6C5, sc-32233; 1:2000), BiP/GRP78 (A-10, sc-376768; 1:1000) and c-Myc (A-14, sc-789, used at 1:500; 9E10, sc-40, used at 1:1000 ) were from Santa Cruz Biotechnology. Anti-cleaved Casp-3 (Asp175) (5A1E, 9664; 1:1000) antibody was from Cell Signaling Technology. Anti-glycogen IgM (1:500) was a generous gift from Dr Otto Baba (Baba, 1993; Nakamura-Tsuruta et al., 2012). Secondary antibodies used in this study were from the following sources: fluorescent-dye-conjugated secondary antibodies (CF-488, CF-568 and CF-647; 1:500) were from Biotium. Anti-rat IgG Alexa Fluor 488 (A-21208; 1:1000) was from Invitrogen and anti-IgM Alexa Fluor 594 (115-585-020; 1:500) was purchased from Jackson Immunoresearch. Horseradish peroxidase (HRP)-conjugated secondary antibodies used for immunoblotting were from Jackson Immunoresearch and were used at a 1:2000 dilution.

### Immunofluorescence staining and microscopy

Cells grown on glass coverslips were fixed with either 4% paraformaldehyde (PFA) in phosphate buffered saline (PBS), pH 7.4, or ice-cold methanol for 10 min at room temperature or −20°C, respectively. PFA-fixed cells were permeabilized with 0.1% Triton X-100 in PBS for 10 min at room temperature. Blocking was performed with 5% normal goat serum (Biosera) in PBS containing 0.05% Tween 20 (PBST) for 1 h at room temperature. Cells were incubated with primary antibodies diluted in 5% normal goat serum in PBST overnight at 4°C and with secondary fluorescent antibodies for 1 h at room temperature. Nuclei were counterstained with DAPI. Images were obtained on a Leica SP2 confocal microscope, using a 40×oil-immersion objective lens and 2×digital zoom, or an Olympus IX73 inverted microscope using the CellSens imaging software. Quantification of colocalization was performed by calculating the thresholded Manders' colocalization coefficient (MCC) (Manders et al., 1993) using the Fiji plugin Coloc2. Values are expressed as mean±s.e.m. of at least five regions of interest.

### Cell treatments

For the induction of ER stress, C2C12 myoblasts were treated with 2 μg/ml TM (Cayman chemicals), 1 μM TG (Sigma-Aldrich), both dissolved in DMSO, or 5 mM 2-deoxy-D-glucose (2-DG) (Sigma-Aldrich) dissolved in H_2_O for 16, 18 or 8 h, respectively. Cells treated with an equal volume of DMSO served as controls. For the inhibition of IRE1, PERK and ATF6 signalling, C2C12 myoblasts were initially pre-treated for 2 h with 20 μM 4μ8C (Millipore), 1 μM GSK2606414 (Millipore) or 400 μM AEBSF (Sigma-Aldrich), respectively, followed by induction of ER stress with 2 μg/ml TM for 16 h in the presence of the above inhibitors. Controls were incubated with an equal volume of DMSO. For the inhibition of autophagy, C2C12 myoblasts, initially treated with 2 μg/ml TM for 16 h, were incubated with 100 nM BafA1 (Sigma-Aldrich) in glucose-free cell culture medium in the absence of TM for 6 h. Controls were incubated in either high-glucose-containing or glucose-free cell culture medium, supplemented with 10% dialysed FBS (Gibco), in the absence of BafA1.

### α-amylase treatment

For the hydrolysis of glycogen by means of α-amylase treatment, cells grown on glass coverslips were fixed using 4% PFA in PBS, permeabilized with 0.1% Triton X-100 in PBS and incubated with 13 U of α-amylase from human saliva (Sigma-Aldrich) diluted in 20 mM phosphate buffer (pH 6.9) at 37°C for 30 min. Controls were fixed and permeabilized as described above but were subsequently treated with an equal volume of 20 mM phosphate buffer under the same conditions.

### Transmission electron microscopy

C2C12 myoblasts cultured in 35-mm cell culture dishes were treated with either TM or DMSO for 16 h and fixed *in situ* in 2.5% glutaraldehyde in 0.1 M phosphate buffer (pH 7.2) for 1 h at room temperature. The cells where then detached, pelleted and washed three times with 0.1 M phosphate buffer (pH 7.2). 1% melted agar (Sigma-Aldrich) was added to the pelleted cells, and they were then placed at −20°C for 5 min. The solidified cell pellet–agar block was post-fixed with 1% osmium tetroxide (BDH Chemicals) for 1 h at room temperature, dehydrated through a graded ethanol series, cleared in propylene oxide and embedded in an epon and araldite resin mixture (Agar Scientific). The resin was polymerized at 60°C overnight. Ultrathin sections of 80–100 nm thickness were prepared on a Reichert-Jung UCT ultramicrotome (Leica), mounted on 200-mesh copper grids (Agar Scientific) and stained with uranyl acetate and lead citrate. Sections were observed on a JEM 1010 transmission electron microscope (JEOL) equipped with a Mega View III digital camera (Olympus).

### Western blotting

Cells were washed and scraped in ice-cold PBS, centrifuged at 825 ***g*** for 5 min and lysed in 150 mM NaCl, 1% Triton X-100, 0.1% SDS, 50 mM Tris-HCl pH 8.0 containing 1×protease inhibitor cocktail (Sigma-Aldrich) for 30 min on ice. Cell lysates were sonicated on ice (three pulses, 5 s each) and cleared by centrifugation at 13,000 ***g*** for 15 min at 4°C. Protein concentration was determined using the Bicinchoninic Acid (BCA) protein assay (Smith et al., 1985). Equal amounts of protein were resolved on either 4–20% Mini-PROTEAN^®^ TGX™ precast protein gels (for cleaved Casp-3; Bio-Rad), standard 15% (for LC3) or 12% SDS–PAGE gels and transferred onto nitrocellulose membranes (Porablot NCP, Macherey-Nagel). Blocking was performed with 5% non-fat milk in TBST (10 mM Tris-HCl pH 8.0, 150 mM NaCl, 0.05% Tween-20 in H_2_O) for at least 1 h. Membranes were sequentially incubated with primary and HRP-conjugated secondary antibodies diluted in blocking buffer overnight and for 1 h, respectively. Proteins were detected using chemiluminescent substrates (LumiSensor™ HRP Substrate Kit, GenScript) and images were obtained with a BioSpectrum 810 imaging system (UVP). Gapdh protein levels were assayed to evaluate equal loading. Densitometry analysis was performed using ImageJ software by normalizing against Gapdh, which was used as loading control. Values are expressed in arbitrary units (AU) and represent the mean±s.e.m. of three independent experiments.

### shRNA-mediated *Stbd1* silencing

*Stbd1* knockdown in C2C12 myoblasts was performed using a lentiviral shRNA approach as described in Demetriadou et al. (2017). Lentiviral vectors expressing *Stbd1*-specific (shStbd1) or scrambled (shScr) control shRNAs were previously reported (Demetriadou et al., 2017). For the generation of the sh3′UTR Stbd1-knockdown C2C12 myoblasts the following oligonucleotides were used: forward, 5′-CCGGGCAACACTGTTTACATCATACCTCGAGGTATGATGTAAACAGTGTTGCTTTTTG-3′; reverse, 5′-AATTCAAAAAGCAACACTGTTTACATCATACCTCGAGGTATGATGTAAACAGTGTTGC-3′. Selection of cells transduced with recombinant lentiviral vectors was performed using 2.5 μg/ml puromycin.

### Generation of C2C12 myoblasts stably overexpressing *Stbd1*

Lentiviral vectors expressing either *Stbd1* or *GFP* (pCCLsin.cPPT.hPGK.eGFP.Wpre) (Santoni de Sio et al., 2006) were transfected in HEK293T cells, and lentiviral particles were produced as described in Demetriadou et al. (2017).

### Quantification of glycogen

Intracellular glycogen was assessed using the glycogen colorimetric/fluorometric assay kit from Biovision (K646). Cells grown on two 100-mm plates were washed and scraped in ice-cold PBS, pelleted by centrifugation at 1670 ***g*** for 5 min and resuspended in 300 μl ice-cold PBS. Where indicated, the cells were treated with either 2 μg/ml TM or DMSO for 16 h prior to harvesting. A 30 μl aliquot of the cell suspension was withdrawn and used for estimation of total protein concentration using the Bicinchoninic Acid (BCA) protein assay (Smith et al., 1985). For the measurement of glycogen, cells were pelleted by centrifugation at 1670 ***g*** for 5 min and lysed in 100 μl ddH_2_O in boiling water for 10 min. The lysate was cleared by centrifugation at 18,000 ***g*** for 10 min and a 10 μl aliquot of each sample was used for the fluorometric determination of glycogen according to the manufacturer’s instructions. Glycogen quantification was performed on the basis of the enzymatic hydrolysis of glycogen to glucose, followed by the oxidation and measurement of the liberated glucose using a fluorescence probe. Fluorescence (excitation 535 nm, emission 587 nm) was measured with a Synergy H1 microplate reader (BioTek). For each sample, fluorescence intensity values were also obtained in the absence of the hydrolysis enzyme and were used to determine and subtract background glucose levels. Glycogen concentration is expressed as μg glucose/mg protein and values represent mean±s.e.m. of duplicate measurements of three independent experiments.

### Establishment of primary myoblast cultures

Cultures of primary myoblasts were established from satellite cells isolated from EDL (extensor digitorum longus) muscles of 8-week-old FVB/N WT mice, as described by Rosenblatt et al. (1995). All animal experiments were performed according to approved guidelines. Single myofibres were incubated in activation medium [high-glucose DMEM with 110 mg/ml sodium pyruvate supplemented with 10% FBS, 2% glutamine, 1% penicillin-streptomycin solution and 0.5% chicken embryo extract (MP Biomedicals)] in 50-mm dishes (Sterilin) treated with 1 mg/ml Matrigel (BD Biosciences) for two days at 37°C, 5% CO_2_ to allow satellite cells to dissociate from myofibres. To enrich for satellite cells over fibroblasts present in the culture, cells were detached using trypsin, re-plated in a non-treated cell culture plate and incubated at 37°C under 5% CO_2_ for 20 min to allow for fibroblasts to attach. Satellite cells contained in the cell culture medium were transferred to Matrigel-treated 6-well plates (SPL Life Sciences) containing proliferation medium [high-glucose DMEM supplemented with 20% FBS, 10% horse serum, 2% glutamine, 1% penicillin-streptomycin solution, 1% chicken embryo extract (MP Biomedicals) and 2.5 ng/ml murine FGF-basic (Peprotech)].

### RNA isolation, reverse transcription and qPCR

Total RNA was extracted using RNAzol^®^RT (Sigma-Aldrich) and employed for first-strand cDNA synthesis using the PrimeScript™ RT Reagent Kit (Perfect Real Time; TaKaRa Bio), following the manufacturers’ instructions. qPCR was performed on an Applied Biosystems QuantStudio™ 7 Flex real-time PCR system using SYBR™ Green PCR Master Mix (Applied Biosystems). Primers used for qPCR are listed in Table S2. Reaction conditions were as follows: 95°C for 3 min, 40 cycles of 95°C for 5 s and 60°C for 25 s followed by a post-amplification melting curve. Raw data were analysed using LinRegPCR version 2020.0 (Ruijter et al., 2009). Values were normalized against *Gapdh* and expressed as the mRNA starting concentration in arbitrary units (AU), corresponding to the mean±s.e.m. of at least three independent experiments.

### Statistical analysis

Statistical significance was determined by one-tailed unpaired Student's *t*-test using the GraphPad Prism 8 software. *P* values ≤0.05 were considered to be statistically significant.

## Supplementary Material

Supplementary information

Reviewer comments
