# Supplementary material for: Stbd1 promotes glycogen clustering during endoplasmic reticulum stress and supports survival of mouse myoblasts
Source: J Cell Sci. 2020 Oct 26;133(20):jcs244855. doi: 10.1242/jcs.244855 (PMC7648618; doi:10.1242/jcs.244855)
Supplement: Supplementary information [file joces-133-244855-s1.pdf]

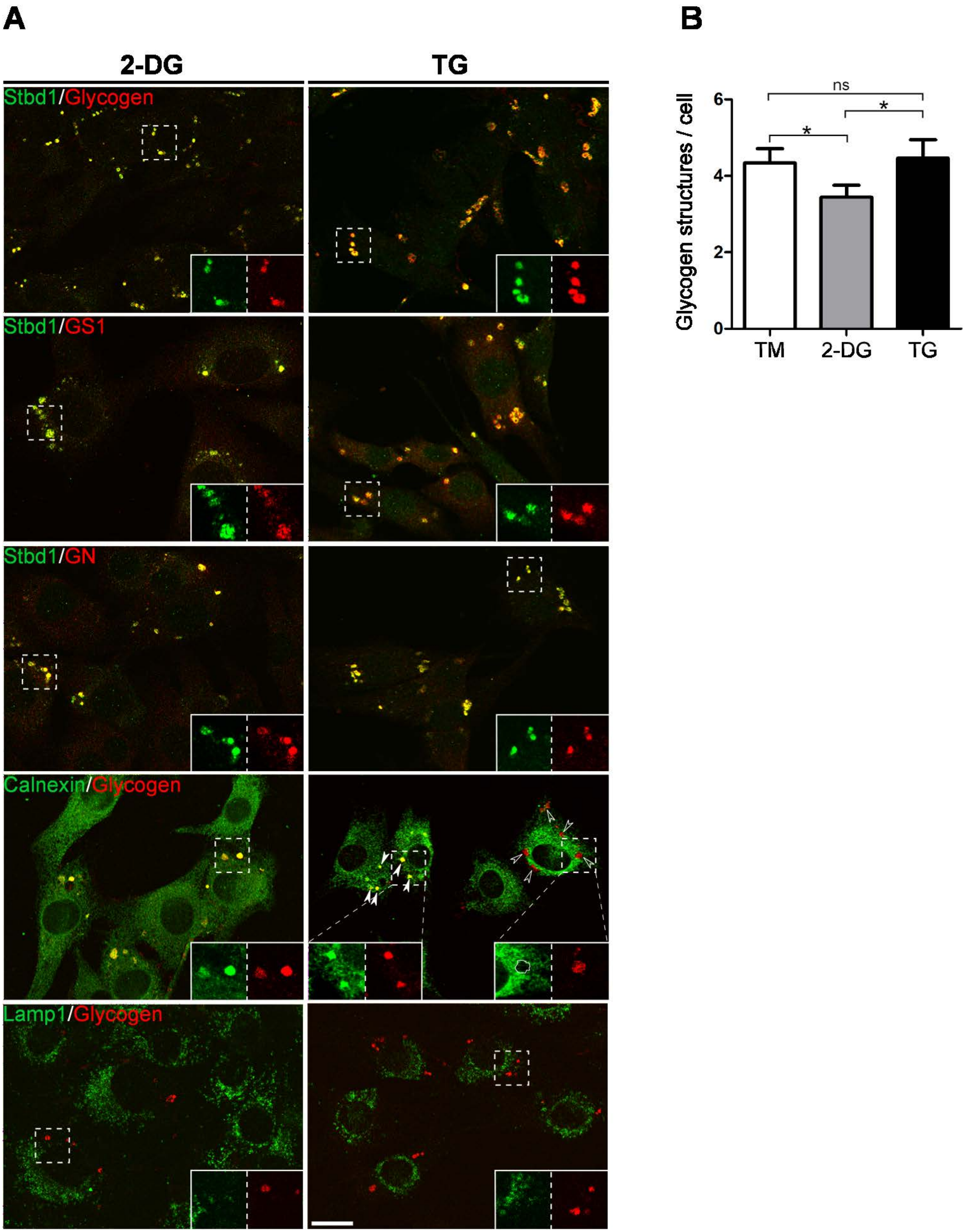

Journal of Cell Science • Supplementary information

Fig. S1. Formation of glycogen-containing, ER clusters is a general response of C2C12 myoblasts to ER stress. (A) C2C12 myoblasts were treated with 5 mM 2-DG (left) or 1 µM TG (right) for 8 or 18 h, respectively, and double stained with the indicated antibodies. Overlays of representative images are shown. Single stainings are shown as inserts and represent higher magnifications of each corresponding boxed area. Both 2-DG and TG induce the formation of glycogen structures which stain positive for Stbd1 [mean±s.e.m. Manders' colocalization coefficient (MCC): 2-DG: 0.760±0.023, *n*=10; TG: 0.789±0.04, *n*=10] GS1 (MCC: 2-DG: 0.823±0.015, *n*=10; TG: 0.752±0.022, *n*=10) and GN (MCC: 2-DG: 0.765±0.021, *n*=10; TG: 0.780±0.015, *n*=10) but not Lamp1 (MCC: 2-DG: 0.080±0.020, *n*=10; TG: 0.068±0.015, *n*=10) thus highly resembling those induced by TM. An inconsistency was noted for glycogen structures induced by TG, some of which stained positive for calnexin (closed arrowheads, MCC: 0.800±0.015, *n*=10) whereas others appeared as calnexin-negative (open arrowheads, MCC: 0.150±0.015, *n*=10). 2-DG-induced glycogen structures displayed positive immunostaining for calnexin (MCC: 0.860±0.008, *n*=10). (B) Average number of glycogen structures formed per cell upon ER stress activation with different ER stress inducers. TM and TG treatment resulted in a comparable number of glycogen clusters per cell (mean±s.e.m., TM: 4.3±0.4, *n*=30; TG: 4.5±0.5, *n*=28) whereas cells treated with 2-DG displayed a smaller average number of structures (mean±s.e.m., 3.4±0.3, *n*=25 clusters/cell). **P*≤0.05; ns, not significant (one-tailed unpaired Student's *t*-test). Scale bar: 20 µm.

Journal of Cell Science • Supplementary information

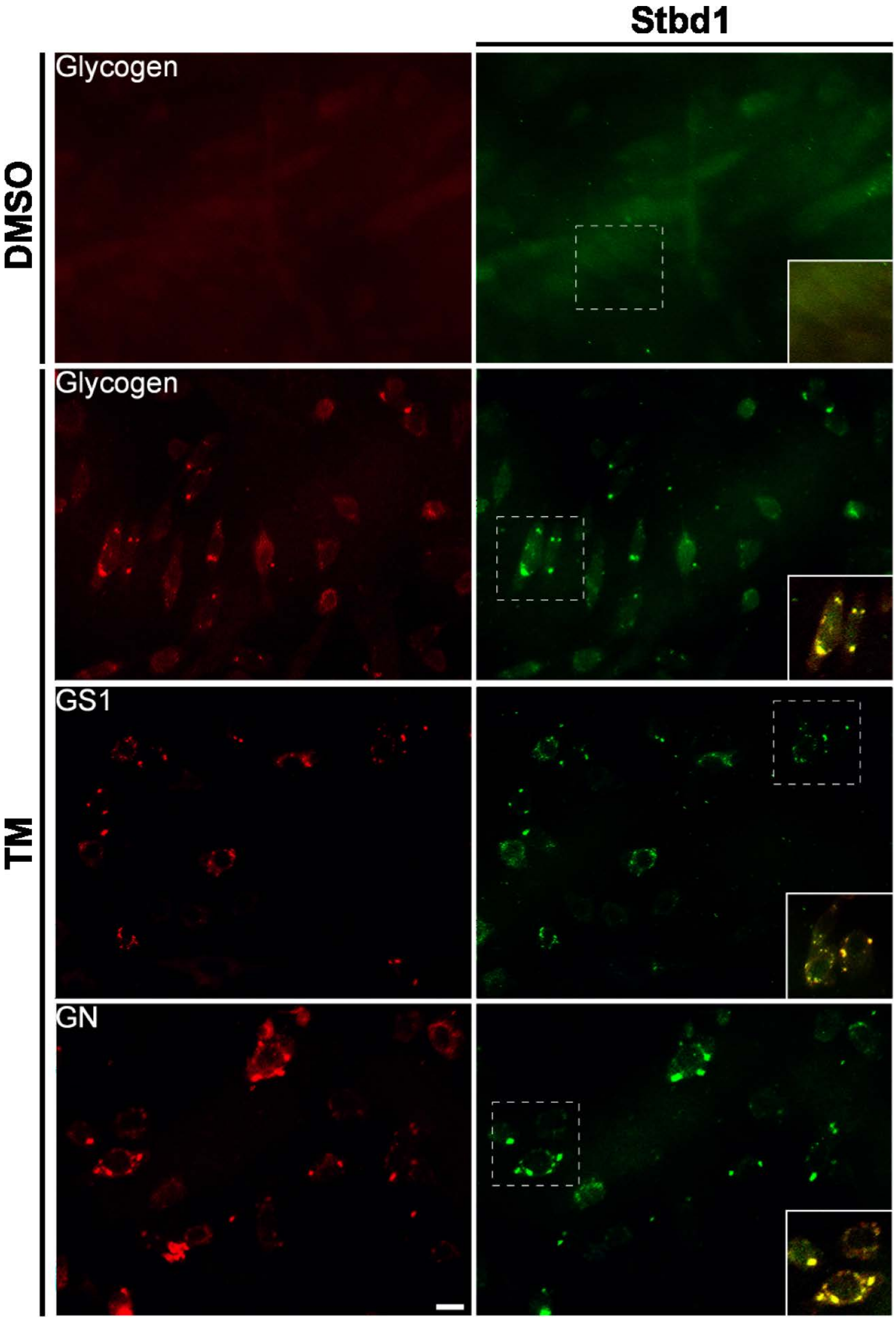
Stbd1
DMSO
Glycogen
TM
Glycogen
GS1
GN

Fig. S2. ER stress induces the formation of glycogen structures in primary mouse myoblasts. Representative immunofluorescent images of primary mouse myoblasts treated with TM or DMSO, vehicle, for 16 h and double-stained with antibodies against Stbd1 (green) and glycogen, GS1 or GN (red). ER stress activation by TM, results in the build-up of glycogen structures that strongly coincide with Stbd1 [mean±s.e.m. Manders' colocalization coefficient (MCC): 0.662±0.037, *n*=10], GS1 (MCC: 0.534±0.169, *n*=10) and GN (MCC: 0.587±0.186, *n*=10). Overlays corresponding to each image pair are shown as inserts and represent higher magnifications of the dashed line boxes. Scale bar: 20 µm.

Journal of Cell Science • Supplementary information

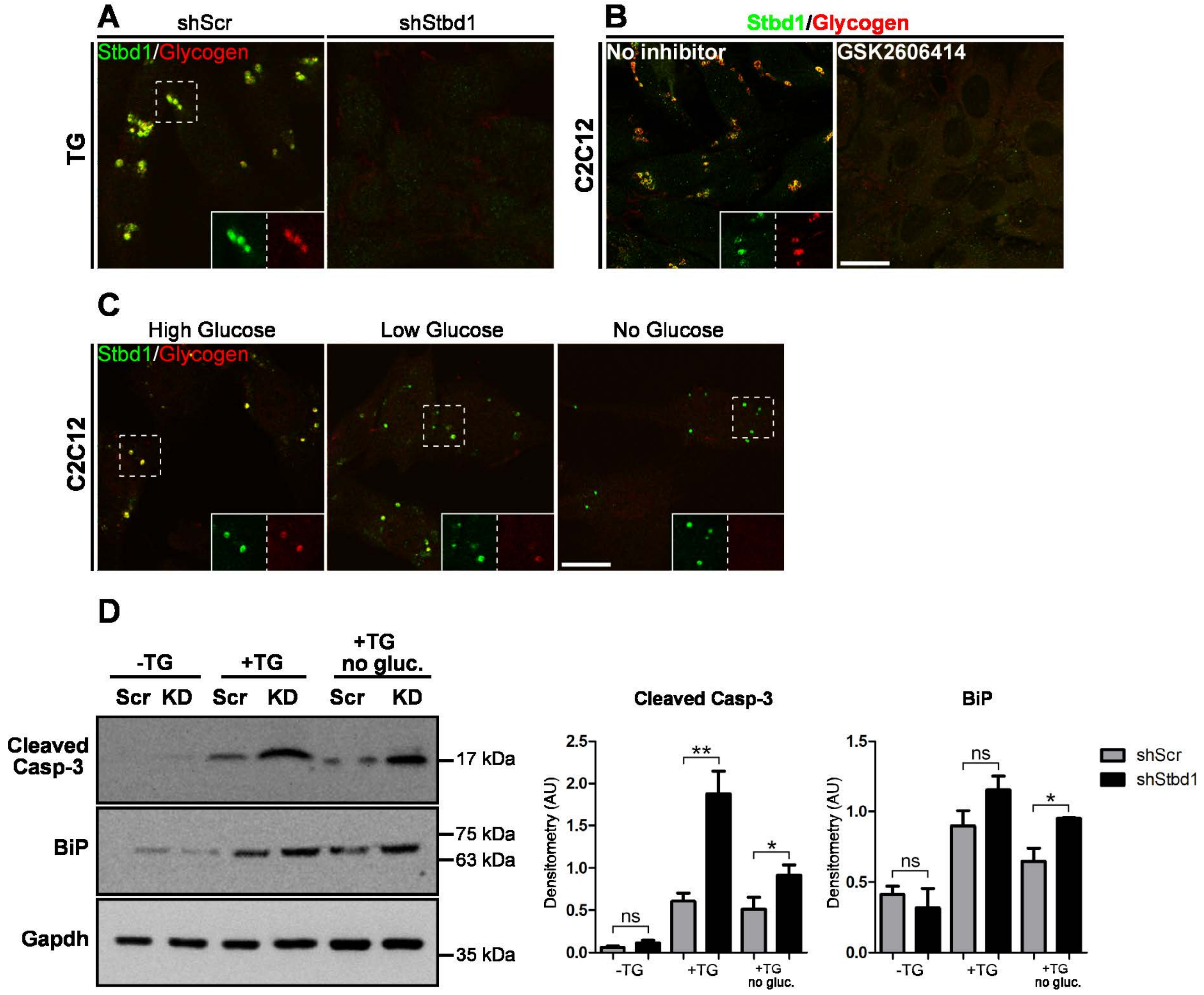

Fig. S3. ER stress activation by TG exerts similar effects as TM in C2C12 myoblasts. (A) Representative double immunofluorescence staining for Stbd1 and glycogen in shScr and shStbd1 cells treated with TG for 18 h. ER stress activation by TG induces the formation of Stbd1-positive glycogen structures in shScr control cells [mean±s.e.m. Manders' colocalization coefficient (MCC): 0.847±0.011, *n*=10] but not in shStbd1 myoblasts. (B) ER stress-induced glycogen structures fail to form in C2C12 myoblasts treated with

Journal of Cell Science • Supplementary information

TG in the presence of the PERK inhibitor GSK2606414. Representative double immunofluorescence staining for Stbd1 and glycogen in C2C12 cells treated with TG in the absence (No inhibitor) or presence of the PERK inhibitor (GSK2606414). (C) C2C12 myoblasts were treated with TG for 18 h, cultured for an additional 6 h without TG in cell culture medium containing either high (25 mM), low (5 mM) or no glucose and stained for glycogen and Stbd1. Representative images are shown. Glycogen is resolved under conditions of glucose restriction whereas ER stress-induced structures still exhibit positive immunostaining for Stbd1 [MCC: high glucose: 0.784±0.014, *n*=10; low glucose: 0.624±0.037, *n*=10; no glucose: 0.061±0.010, *n*=10]. Inserts in A, B and C represent higher magnification of single stainings of the corresponding boxed areas. (C) Western blot (left) and densitometry (right; mean±s.e.m, *n*=3) on lysates from shScr and shStbd1 (KD) C2C12 cells at basal culturing conditions (-TG), after 18 h of TG treatment (+TG) or after 18 h of TG treatment followed by TG withdrawal and additional culturing of cells for 6 h in glucose-free medium (+TG no gluc.). TG treatment resulted in significantly elevated protein levels of cleaved Casp-3 in KD cells compared to shScr controls and these remained significantly increased even after the removal of TG. ER stress activation by TG was confirmed by monitoring the protein levels of BiP and Gapdh was used as a loading control. *P≤0.05; **P≤0.01; ns, not significant (one-tailed unpaired Student's *t*-test). Scale bars: 20 µm.

Journal of Cell Science • Supplementary information

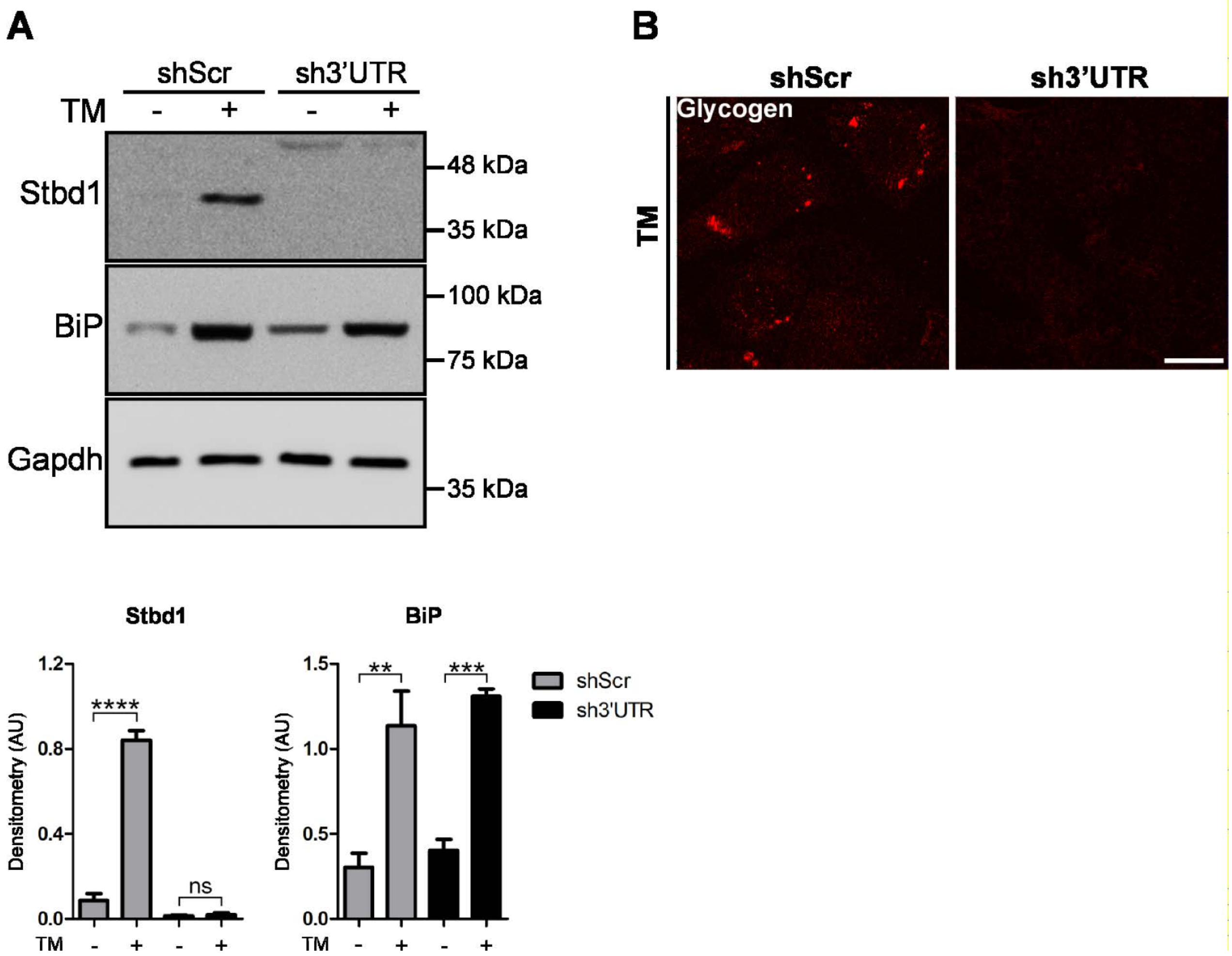

Fig. S4. Characterization of sh3'UTR Stbd1 knockdown C2C12 myoblasts. (A) Western immunoblot (top) and densitometry (bottom; mean±s.e.m, *n*=3) for the assessment of Stbd1-silencing efficiency. Protein extracts from C2C12 myoblasts expressing either a scrambled (shScr) or a Stbd1-specific (sh3'UTR) shRNA sequence were prepared in the absence (-) or presence (+) of TM and probed with an antibody against Stbd1. In contrast to shScr cells ER stress-induced Stbd1 upregulation does not occur in sh3'UTR cells. BiP antibody was used to validate UPR activation upon TM treatment. Gapdh is shown as a loading control. (B) Representative immunofluorescence staining for glycogen in TM-treated shScr and sh3'UTR C2C12 cells. Glycogen structures fail to form in sh3'UTR Stbd1 knockdown cells. ***P*≤0.01; ****P*≤0.001; *****P*≤0.0001; ns, not significant (one-tailed unpaired Student's *t*-test). Scale bar: 20 µm.

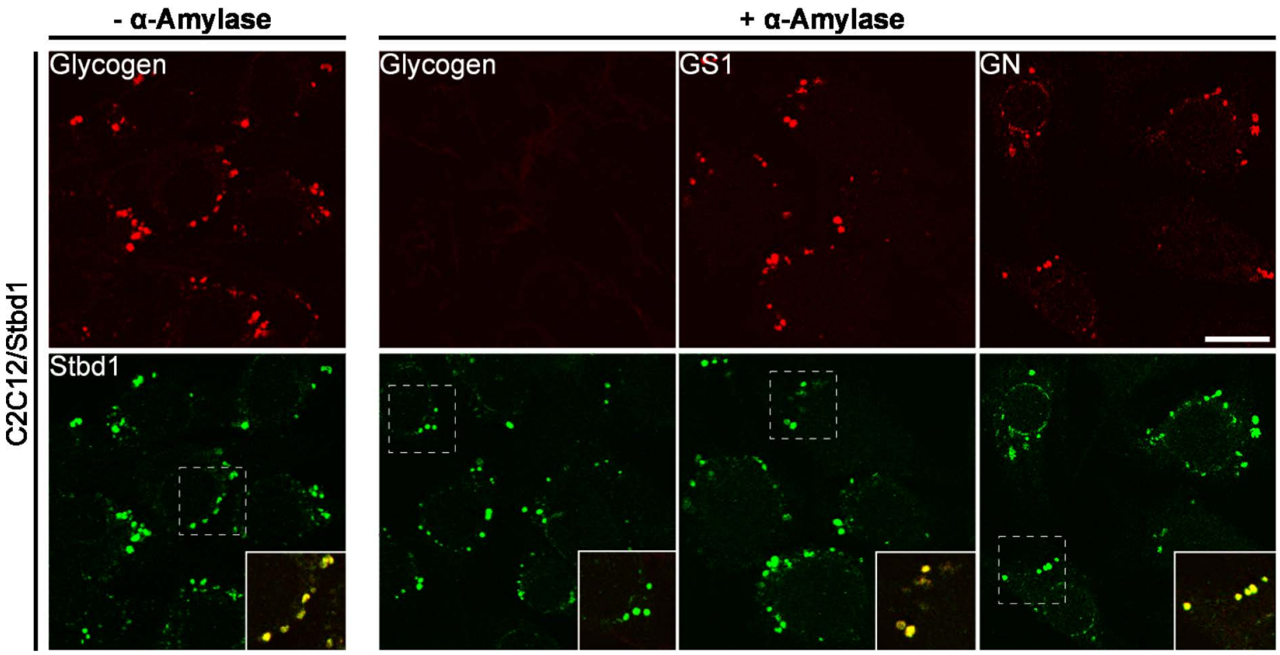

Fig. S5. Stbd1 overexpression results in the formation of structures containing α-amylase-degradable glycogen. C2C12 cells stably overexpressing Stbd1 were incubated in the absence (-α-amylase) or presence (+α-amylase) of α-amylase and double-stained for Stbd1 (green) and glycogen [mean±s.e.m. Manders' colocalization coefficient (MCC): -α-amylase: 0.819±0.024, *n*=10; +α-amylase: 0.136±0.021, *n*=10], GS1 (MCC: 0.806±0.022, *n*=10) or GN (MCC: 0.800±0.013, *n*=10). Representative overlay images of the corresponding boxed areas are shown as inserts. Treatment with α-amylase results in the complete degradation of glycogen without affecting the presence of Stbd1, GS1 and GN on intracellular assemblies. Scale bar: 20 µm.

Table S1. Rescue efficiency of different Stbd1 variants

Stbd1 variant	TM	Number of cells counted		Rescue (%)
		Myc (+) / Glycogen (+)	Myc (+) / Glycogen (-)	
Stbd1-Myc (WT)	-	84	24	**77.8** (*n*=108)
	+	117	10	**92.1** (*n*=127)
W188A/V191A-Myc	-	103	38	**73.0** (*n*=141)
	+	131	18	**87.9** (*n*=149)
W273G-Myc	-	0	48	**0** (*n*=48)
	+	0	46	**0** (*n*=46)

Journal of Cell Science • Supplementary information

Table S2. Primers used for qPCR

Gene	Primer sequence
Atf4	F: 5’-ATGGCCGGCTATGGATGAT-3’<br>R: 5’-CGAAGTCAAACTCTTTCAGATCCATT-3’
BiP	F: 5’-ACTCCGGCGTGAGGTAGAAA-3’<br>R: 5’-AGAGCGGAACAGGTCCATGT-3’
Chop	F: 5’-CCACCACACCTGAAAGCAGAA-3’<br>R: 5’-AGGTGCCCCCAATTTCATCT-3’
sXbp1	F: 5’-ACACGCTTGGGAATGGACAC-3’<br>R: 5’-CCATGGGAAGATGTTCTGGG-3’
Ppp1r3c	F: 5’-TGAGCTGCACCAGAATGATCC-3’<br>R: 5’-GGTGGTGAATGAGCCAAGCA-3’
Gapdh	F: 5’-TGACGTGCCGCCTGGAGAAA-3’<br>R: 5’-AGTGTAGCCCAAGATGCCCTTCAG-3’
F: forward, R: reverse	

Journal of Cell Science • Supplementary information
