# Supplementary material for: Stbd1 promotes glycogen clustering during endoplasmic reticulum stress and supports survival of mouse myoblasts
Source: J Cell Sci. 2020 Oct 26;133(20):jcs244855. doi: 10.1242/jcs.244855 (PMC7648618; doi:10.1242/jcs.244855)
Supplement: Reviewer comments [file joces-133-244855_review_history.pdf]

Stbd1 promotes glycogen clustering during endoplasmic reticulum stress and supports survival of mouse myoblasts

Andria A. Lytridou, Anthi Demetriadou, Melina Christou, Louiza Potamiti, Nikolas P. Mastroyiannopoulos, Kyriacos Kyriacou, Leonidas A. Phylactou, Anthi Drousiotou and Petros P. Petrou
DOI: 10.1242/jcs.244855

Editor: Jennifer Lippincott-Schwartz

Review timeline

Original submission:	3 February 2020
Editorial decision:	30 March 2020
First revision received:	17 August 2020
Accepted:	16 September 2020

Original submission

First decision letter

MS ID#: JOCES/2020/244855

MS TITLE: Stbd1 promotes glycogen aggregation during endoplasmic reticulum stress and supports survival of mouse myoblasts

AUTHORS: Andria A. Lytridou, Anthi Demetriadou, Melina Christou, Louiza Potamiti, Nikolas P Mastroyiannopoulos, Kyriacos Kyriacou, Leonidas A Phylactou, Anthi Drousiotou, and Petros P Petrou
ARTICLE TYPE: Research Article

We have now reached a decision on the above manuscript.

To see the reviewers' reports and a copy of this decision letter, please go to: https://submit-jcs.biologists.org and click on the 'Manuscripts with Decisions' queue in the Author Area. (Corresponding author only has access to reviews.)

As you will see, the reviewers raise a number of substantial criticisms that prevent me from accepting the paper at this stage. They suggest, however, that a revised version might prove acceptable, if you can address their concerns. If you think that you can deal satisfactorily with the criticisms on revision, I would be pleased to see a revised manuscript. We would then return it to the reviewers.

We are aware that you may currently be unable to access the lab to undertake experimental revisions. If it would be helpful, we encourage you to contact us to discuss your revision in greater detail. Please send us a point-by-point response indicating where you are able to address concerns raised (either experimentally or by changes to the text) and where you will not be able to do so within the normal timeframe of a revision. We will then provide further guidance. Please also note that we are happy to extend revision timeframes as necessary.

Please ensure that you clearly highlight all changes made in the revised manuscript. Please avoid using 'Tracked changes' in Word files as these are lost in PDF conversion.

© 2020. Published by The Company of Biologists under the terms of the Creative Commons Attribution License (https://creativecommons.org/licenses/by/4.0/).

I should be grateful if you would also provide a point-by-point response detailing how you have dealt with the points raised by the reviewers in the 'Response to Reviewers' box. Please attend to all of the reviewers' comments. If you do not agree with any of their criticisms or suggestions please explain clearly why this is so.

Reviewer 1

Advance summary and potential significance to field

In their paper entitled "Stbd1 promotes glycogen aggregation during endoplasmic reticulum stress and supports survival of mouse myoblasts," Lytridou et al. demonstrate that ER stress induces the expression of glycogen-binding protein Stbd1 in mouse myoblasts to promote glycogen aggregation. Interestingly, these glycogen aggregates are degraded under glucose depleted conditions in autophagy/lysosome-independent manner. The authors further propose that failure to induce glycogen aggregation during ER stress, as is the case in Stbd1 knockdown cells, results in increased apoptosis. However, the cellular advantage for glycogen accumulation and clearance under ER stress was unclear. While it was clearly shown that ER stress induces Stbd1 expression and that Stbd1 is required for the formation of glycogen aggregates under these conditions, the impact of this study could be improved by addressing the following points:

Comments for the author

1. The authors need to provide quantitation (colocalization, frequency etc.) for all immunofluorescence images and western blots (Figs 1A,C; 2; 3; 4A,C,E,F; 5A,B; 6B,C; 7; 8A,C.) to make solid conclusions based on their observations. Additionally, control images should be included for each experiment.
2. Several times in the manuscript, the authors elude to the possibility of Stbd1 acting as a scaffold protein for the recruitment and direct binding of other proteins such as GN and GS1. This statement might be confirmed through biochemical evidence of direct binding in C2C12 myoblasts, or removed.
3. It is interesting that Stbd1 is required for ER stress-induced glycogen aggregation (Fig. 4C). Can this effect be rescued by overexpressing wild type Stbd1 or a Stbd1 mutant that is unable to bind to glycogen? Further, to strengthen the mechanistic claim that loss of glycogen aggregation in Stbd1 depleted cells increases ER stress-induced cell death (Fig. 8C), the authors could perform rescue experiments with WT and glycogen-binding deficient Stbd1 mutants.
4. It is intriguing that induction of Stbd1 and glycogen granule accumulation occurs specifically downstream of PERK signaling. The authors could further explore this observation and provide more mechanistic insight into how this PERK specificity occurs, perhaps through a PERK knockdown examination of downstream transcriptional targets, and perturbation of signaling intermediates.
5. The loss of glycogen aggregates in glucose deprived cells is another striking finding. However, loss of the aggregates is autophagy independent (based on Bafilomycin A1 treatment). The authors might complement these findings with Atg5/7 knockdown which would specifically block autophagosome biogenesis. Additionally, is Atg8 family interacting motif (AIM) of Stbd1 required for glycogen aggregate formation and/or clearance of these aggregates under low glucose conditions? Does glycogen clearance require functional glycogen phosphorylase enzyme that catalyzes the breakdown of glycogen to glucose under these conditions?
6. Further, an examination of cellular energetics and the implication of mitochondria (as implicated in the discussion) may provide interesting insight into the correlation between glycogen aggregates and cell survival.
7. While the authors demonstrate the use of alternative ER stressors and cell models show a similar phenotype to their initial observation (without quantitative evidence), key experiments such as definitive PERK signaling remediation in response to glucose, and the impact on cell survival should be repeated with other mechanisms of ER stress induction and primary myoblasts.

© 2020. Published by The Company of Biologists under the terms of the Creative Commons Attribution License (https://creativecommons.org/licenses/by/4.0/).

Reviewer 2

Advance summary and potential significance to field

In this manuscript, these authors report that myoblasts, in response to treatment with Tunicamycin (TM) for 16 h, have significantly increased levels of Stbd1 and glycogen aggregate formation. These glycogen aggregates colocalize with Stbd1, GS1, GN and calnexin and are α-amylase degradable. They also show that whereas shRNA-mediated Stbd1 knockdown appears to abolish Stbd1 expression and the associated glycogen aggregate formation, in the presence of TM, this does not impact cell glycogen levels. This effectively separates the role of Stbd1 in TM-induced ER-associated glycogen aggregate formation from glycogen synthesis.

Stbd1-mediated formation of ER-associated glycogen aggregates in response to ER stress could have far-reaching implications with regards to the relationship between ER stress and metabolism, which is likely critical during periods of myoblast differentiation and skeletal muscle development; but with the potential for a broader impact on other tissues.

Comments for the author

Reviewer Report

This manuscript describes a set of interesting observations related to the role of Stbd1 in glycogen aggregate formation in response to ER stress induced by tunicamycin (TM). The conclusion of Stbd1- dependent aggregate formation in C2C12 myoblasts in response to TM is well supported by the data and can be published on its own (e.g. as a short report) after addressing my first major concern, and with only minor other revisions. However, I have major concerns regarding other observations and conclusions. If the authors decide to keep them in their revised manuscript, these concerns should be addressed.

Major concerns (require additional experiments)

1. It is unclear whether the Stbd1-induced glycogen aggregates are cytosolic or perhaps a collection of granules encapsulated by lysosomes, which may occur during glycogenolysis. Although unlikely, a 16h tunicamycin-induced increase in cell glycogen levels could also be due to defective degradation. The TEM images display cytosolic aggregates, but together with the data in Figure 8 it is still not enough to eliminate this possibility. I recommend either repeating key experiments with lysosome markers (anti- Lamp1, Lysotracker) or presenting a much more cautious interpretation of the results citing a lack of membrane enveloping aggregates in TEM as evidence in favor of cytosolic aggregates.

2. It is unclear whether the difference between the effects of N-glycosylation inhibitors (TM and 2DG) and the SERCA (Ca-flux) inhibitor (TG) is due to their effects related to ER stress (as presumed by the authors) or due to their primary mechanisms of action listed above. Note that the ER stress is their secondary rather than primary effect. Therefore, selective effects of N-glycosylation inhibitors cannot be interpreted as ER stress effects without demonstrating similar action of unrelated agents inducing ER stress (e.g, Brefeldin A, DTT, etc). An alternate approach is to limit the scope of the paper to tunicamycin-induced effects rather than ER stress effects.

3. It is unclear why the authors switch from myoblasts to MEFs for PTG experiments. With such effective shRNA Stbd1 KD, experiments in cells previously characterized (i.e. C2C12 myoblast cell line or primary myoblasts) should be provided with some interpretation to justify the need for the new cell line. In that case, the new cell line (MEFs) would have to be fully characterized with: (a) IF of GS1, GN and glycogen; (b) glycogen levels; (c) α-amylase degradation; (d) Stbd1-overexpression; and (e) all additional experiments that classify these aggregates as ER-associated or cytosolic.

4. It is unclear whether PTG levels also increase with TM or Stbd1 overexpression, whether the glycogen aggregates induced by PTG also colocalize with GS1/GN, or whether these aggregates are in lysosomes. An alternate approach (for both concern #3 and #4) is to limit the scope of the paper to ER-associated glycogen aggregates induced by TM/Stbd1-overexpression in myoblasts.

© 2020. Published by The Company of Biologists under the terms of the Creative Commons Attribution License (https://creativecommons.org/licenses/by/4.0/).

5. It is unclear how the authors can assess impacts related to inhibition of IRE1, ATF6 and PERK without proper readouts to confirm activity and specificity of the corresponding inhibitors in their cells under the conditions of their experiments. I recommend either additional experiments or much more cautious discussion of the effects of the inhibitors.

Minor comments and suggested revisions
a. The manuscript would be strengthened by the presentation of a cohesive model and some introduction of glycogen and glycogen granules, how glycogen granules differ (if they do) from these larger glycogen clusters, and the mechanisms of glycogenesis and glyocogenolysis.

b. WB quantifications would be useful.

c. If available, for consistency, please include intracellular glycogen levels for all experiments to separate glycogen cluster formation/breakdown from glycogenesis/glycogenolysis.

d. The manuscript would be strengthened by quantification of glycogen clusters (i.e average number of clusters/cell) and the change with condition.

e. The glycogen clusters are referred to as structures, clusters, aggregates, ER aggregates, inclusions, granules and assemblies. As the use of some of these terms may indicate a particular biochemical composition (i.e. aggregates), perhaps limit the variable terms to structures or clusters. The term ‘ER aggregates’ is especially confusing as it traditionally refers to protein aggregates that form within the ER lumen.

f. In Fig. 1, the authors characterize the TEM images with the claim “TM treatment induced the formation of glycogen-containing ER aggregates (F, arrow). Representative examples of membrane- associated glycogen granules are shown by arrowheads in G…” It is unclear how the TEM displays membrane-associated glycogen assemblies. How are these being characterized?

g. The interpretation of Fig. 1 E is questionable. In the TEM image, the cell appears extremely unhealthy, with large amounts of blebbing, making it very difficult to resolve organelles (especially ER). As a fixative composed of 2.5%gluteraldehyde in 0.1M phosphate buffer instead of the more traditional 0.1M sodium cacodylate was used, I am unsure whether the indicated regions of dilated ER are in fact dilated ER, or simply ruptured organelles (e.g. mitochondria) or another fixation-related artifact.

h. Please provide associated calnexin stain for Figure 6C, if this experiment is retained in the revised manuscript.

i. It is unclear why Chop nuclear translocation was used to indicate ER stress induced by TM in MEFs (Fig. S3), but an increase in BiP was used elsewhere (Figure 4).

First revision

<u>Author response to reviewers' comments</u>

Please see also PDF file entitled "Response to Reviewers comments", in Supplementary Information

MS ID#: JOCES/2020/244855
MS TITLE: Stbd1 promotes glycogen aggregation during endoplasmic reticulum stress and supports survival of mouse myoblasts
AUTHORS: Andria A. Lytridou, Anthi Demetriadou, Melina Christou, Louiza Potamiti, Nikolas P Mastroyiannopoulos, Kyriacos Kyriacou, Leonidas A Phylactou, Anthi Drousiotou, and Petros P. Petrou
ARTICLE TYPE: Research Article

© 2020. Published by The Company of Biologists under the terms of the Creative Commons Attribution License (https://creativecommons.org/licenses/by/4.0/).

Point-by-point response to Reviewer's comments
Reviewer #1
The Reviewer considers that it is clearly shown in our manuscript that ER stress induces Stbd1 expression and that Stbd1 is required for the formation of glycogen aggregates under these conditions. However, the Reviewer feels that the cellular advantage for glycogen accumulation and clearance under ER stress was unclear.
The author does not raise substantial criticism or major concerns on the quality and the interpretation of the data but provides a number of suggestions which according to the Reviewer could improve the impact of the study.
1. "The authors need to provide quantitation (colocalization, frequency, etc.) for all immunofluorescence images and western blots (Figs 1A,C; 2; 3; 4A,C,E,F; 5A,B; 6B,C; 7; 8A,C.) to make solid conclusions based on their observations."
We provide quantification of colocalization (thresholded Manders' colocalization coefficient) for all immunofluorescence images featuring double immunostainings and detectable specific fluorescence signals. The colocalization coefficients are given in the legend of each Figure, in the revised manuscript.
We further provide quantification and statistical analysis of all western blots in the study, as suggested by the Reviewer.
"Additionally, control images should be included for each experiment."
We find it difficult to follow the above comment made by the Reviewer, since the Reviewer does not specify which experiments in the study require control images. We believe that we provide images and results of controls in all experiments included in the study.

2. "Several times in the manuscript, the authors elude to the possibility of Stbd1 acting as a scaffold protein for the recruitment and direct binding of other proteins such as GN and GS1. This statement might be confirmed through biochemical evidence of direct binding in C2C12 myoblasts, or removed."
A physical interaction between human Stbd1 and glycogen synthase has been previously demonstrated (Zhu, Y. et al., Biosci. Rep. (2014), 34: e00117) by co-immunoprecipitation. In this previously published study it was further shown that, glycogen degradation did not abolish but instead strengthened the above interaction and accordingly the authors of this study concluded that glycogen is not required to bridge this interaction.
Additionally, proteins interacting with Stbd1 have been identified in a large scale proteomic screening (Huttlin, et al., Nature (2017), 505-509). These proteins included glycogenin (GN)-1 and -2 and also glycogen synthase (GS)-1 and -2. The above study further confirmed previously characterized interactions of Stbd1 with Laforin, glycogen debranching enzyme and Gabarpl1.
Our data are in agreement with the aforementioned published findings. We show that GS1 and GN colocalize with Stbd1 on glycogen structures formed both in response to ER stress activation and Stbd1 overexpression and that this does not occur in the absence of Stbd1. Most importantly when glycogen is degraded by α-amylase treatment, both GS1 and GN remain colocalized with Stbd1 further supporting direct interactions between the proteins not bridged by glycogen (revised Fig. 2 and Fig. S5).
We feel that carrying out in vitro co-precipitation experiments would basically repeat and confirm previously published findings and would not significantly increase the impact of our study. However, to comply with the Reviewer's comment we have formulated statements made in the text with regards to the above in a more cautious way:
o Line 153-155: "…..depletion of glycogen may indicate that during ER stress, the above proteins form a complex with Stbd1 at the ER membrane through direct protein-protein interactions not mediated by glycogen."
o Line 274-275: "……supports that Stbd1 may form a scaffold at the ER membrane mediating the recruitment of GS1 and GN likely through direct protein-protein interactions."
o Line 431-432: "….suggesting that Stbd1 may serve as a scaffold mediating the recruitment of GS1 and GN to the ER membrane through direct protein-protein interactions."

3. "It is interesting that Stbd1 is required for ER stress-induced glycogen aggregation (Fig. 4C). Can this effect be rescued by overexpressing wild type Stbd1 or a Stbd1 mutant that is unable to bind to glycogen?"
We thank the Reviewer for pointing out this direction. For the generation of the Stbd1 knockdown cells reported in the initial version of the manuscript we have used a shRNA sequence which

© 2020. Published by The Company of Biologists under the terms of the Creative Commons Attribution License (https://creativecommons.org/licenses/by/4.0/).

targeted a region within the coding sequence of the cDNA. These cells could not be employed for rescue experiments. However, to address the Reviewer's suggestion we have generated and characterized (revised Fig. S4) a second Stbd1 knockdown C2C12 stable cell line using a target sequence within the 3'UTR of the gene and used these cells (sh3'UTR) for rescue experiments.
As suggested by the Reviewer, we have performed rescue experiments with wild type (WT) Stbd1 and the W273G glycogen binding-deficient mutant (Demetriadou et al., J. Cell Sci. (2017), 130:903-915). In addition we have also generated a Stbd1 variant carrying mutations in two conserved residues (W188A/V191A) within the AIM motif (see Reviewer #1, point 5) previously shown for human Stbd1 to abolish its interaction with Gabarapl1 (Jiang, S. et al., Biochem. Biophys. Res. Commun. (2011) 413, 420-425).
We show that both WT Stbd1 and the AIM mutant but not the glycogen-binding deficient variant rescue glycogen clustering already in the absence of ER stress. Results are shown in revised Fig. 3E and Table S1.

"Further, to strengthen the mechanistic claim that loss of glycogen aggregation in Stbd1 depleted cells increases ER stress-induced cell death (Fig. 8C), the authors could perform rescue experiments with WT and glycogen-binding deficient Stbd1 mutants."
We thank the Reviewer for the insightful suggestion. We have attempted to address the Reviewer's comment by stably overexpressing the WT and W273G glycogen-binding deficient Stbd1 mutant in sh3'UTR, Stbd1 knockdown cells, by lentiviral infection. We found that although we could achieve overexpression of WT Stbd1 this was not the case for the W273G mutant. This result confirms a previous observation made with the corresponding human W293G glycogen binding deficient variant which was found to be unstable and degraded by the proteasome in stable cell lines (Zhu, Y. et al., Biosci. Rep. (2014), 34: e00117). Due to the lack of the W273G negative control a rescue of ER-stress induced cell death could not be reliably assessed.

4. "It is intriguing that induction of Stbd1 and glycogen granule accumulation occurs specifically downstream of PERK signaling. The authors could further explore this observation and provide more mechanistic insight into how this PERK specificity occurs, perhaps through a PERK knockdown, examination of downstream transcriptional targets, and perturbation of signaling intermediates"
Our data demonstrate that the PERK specific inhibitor GSK2606414 inhibits ER stress-induced Stbd1 upregulation and prevents the formation of glycogen structures. It is well established that the above inhibitor is highly selective for PERK (Axten, J. M. et al., J. Med. Chem. (2012), 55, 7193-7207). We therefore believe that a PERK knockdown will have the same effect as the inhibitor. Moreover, we hope that the Reviewer shares our view that the molecular/mechanistic insights of Stbd1 activation through the PERK signalling pathway is beyond the scope of the current study since this would require promoter analysis, identification of transcription factor binding sites etc. However, we recognize that we do not demonstrate specificity of the inhibitors used and their effects on downstream UPR targets and we thank the Reviewer for pointing this out. To address the Reviewer's suggestion we provide data of the action of the three inhibitors used (4μ8C, AEBSF and GSK2606414) on the expression of representative target genes (spliced Xbp1, Atf4, BiP and Chop) which also includes downstream targets of PERK. Data are shown in revised Fig. 4A.
5. "The loss of glycogen aggregates in glucose deprived cells is another striking finding. However, loss of the aggregates is autophagy independent (based on Bafilomycin A1 treatment). The authors might complement these findings with Atg5/7 knockdown which would specifically block autophagosome biogenesis."
The above, as well as additional related points raised below by the Reviewer with regards to the mechanism of clearance of ER stress-induced glycogen structures, are indeed very interesting and worth investigating. However, studying the molecular/mechanistic details could provide a separate future study by itself. We hope that the Reviewer acknowledges that the generation and characterization of all suggested knockdown cell lines was not feasible within the time frame of this revision.
"Additionally, is Atg8 family interacting motif (AIM) of Stbd1 required for glycogen aggregate formation and/or clearance of these aggregates under low glucose conditions?"
We provide evidence (revised Fig. 3E and table S1) that a functional AIM motif is not required for the formation glycogen structures. Cells overexpressing a Stbd1 variant carrying mutations in two conserved residues within the AIM motif (W188A/V191A) display glycogen structures already in the absence of ER stress activation. The above two mutations were previously shown for human Stbd1

© 2020. Published by The Company of Biologists under the terms of the Creative Commons Attribution License (https://creativecommons.org/licenses/by/4.0/).

to compromise its interaction with Gabarapl1 (Jiang, S. et al., Biochem. Biophys. Res. Commun. (2011) 413, 420-425).
"Does glycogen clearance require functional glycogen phosphorylase enzyme that catalyzes the breakdown of glycogen to glucose under these conditions?"
Again the above is a very interesting question to address with regards to the mechanisms of glycogen structure resolution under conditions of glucose restriction. However, we think that this is difficult to address because of the following reasons: since there are three different isoforms of glycogen phosphorylase (liver, muscle and brain) it should first be investigated which of these are expressed in C2C12 cells. Being myoblasts, does not exclude that additional isoforms other than the muscle isoenzyme are expressed in these cells. This is exemplified by the fact that C2C12 cells express both the muscle and liver isoform of glycogen synthase (Pescador, N. et al., Plos One (2010) Mar 12;5(3):e9644). On the other hand, to our knowledge there is no inhibitor which blocks the activity of all three isoforms.
However, we have attempted to address the Reviewer's point by employing an inhibitor of muscle glycogen phosphorylase (KB228, Calbiochem). We found that ER stress-induced glycogen structures still resolve at a concentration of 3μM of the inhibitor, however we cannot draw a safe conclusion since this could be due to the activity of other phosphorylases not blocked by the inhibitor.
6. "Further, an examination of cellular energetics and the implication of mitochondria (as implicated in the discussion) may provide interesting insight into the correlation between glycogen aggregates and cell survival."
We agree with the Reviewer that it is a very interesting point to address. Again we feel, and ask for the Reviewer's understanding, that the study of cellular and mitochondrial energetics and dynamics is beyond the scope of the current manuscript. Investigating the above would require a substantial amount of time and experimentation and cannot be assessed as one of many issues addressed for the purposes of the current revision.
7. "While the authors demonstrate the use of alternative ER stressors and cell models show a similar phenotype to their initial observation (without quantitative evidence), key experiments such as definitive PERK signaling, remediation in response to glucose, and the impact on cell survival should be repeated with other mechanisms of ER stress induction and primary myoblasts."
We thank the Reviewer for the above insightful comment. We have repeated key experiments which had been performed with tunicamycin (TM), using thapsigargin (TG) as an ER stress inducer, as suggested by the Reviewer. TM and TG induce ER stress through different molecular mechanisms (inhibition of protein glycosylation and inhibition of calcium transport into the ER, respectively). Results of experiments using TG are shown in revised Fig. S3 and include:
a) Lack of ER stress-induced glycogen structures in shScr myoblasts (Fig.S3A)
b) Lack of ER stress-induced glycogen structures in the presence of the PERK inhibitor GSK2606414 (Fig. S3B)
c) Resolution of ER stress-induced glycogen under conditions of glucose starvation (Fig. S3C)
d) Increased levels of the activated/cleaved Caspase 3 in TG treated shStbd1 cells (Fig. S3D)
The above results confirm the data obtained with TM.

Reviewer #2
The Reviewer thinks that the manuscript "describes a set of interesting observations related to the role of Stbd1 in glycogen aggregate formation in response to ER stress induced by tunicamycin (TM)." Furthermore the Reviewer considers that:
"The conclusion of Stbd1-dependent aggregate formation in C2C12 myoblasts in response to TM is well supported by the data and can be published on its own (e.g. as a short report) after addressing my first major concern, and with only minor other revisions. However, I have major concerns regarding other observations and conclusions. If the authors decide to keep them in their revised manuscript, these concerns should be addressed."
We believe that we have addressed the Reviewer's concerns and have therefore kept all data included in the initial version of the manuscript.
Major concerns of the Reviewer
1. "It is unclear whether the Stbd1-induced glycogen aggregates are cytosolic or perhaps a collection of granules encapsulated by lysosomes, which may occur during glycogenolysis.
Although unlikely, a 16h tunicamycin-induced increase in cell glycogen levels could also be due to defective degradation. The TEM images display cytosolic aggregates, but together with the data in Figure 8 it is still not enough to eliminate this possibility. I recommend either repeating key experiments with lysosome markers (anti-Lamp1, Lysotracker) or presenting a much more cautious

© 2020. Published by The Company of Biologists under the terms of the Creative Commons Attribution License (https://creativecommons.org/licenses/by/4.0/).

interpretation of the results citing a lack of membrane enveloping aggregates in TEM as evidence in favor of cytosolic aggregates."
We thank the Reviewer for pointing out the above. We have included Lamp1 immunostaining and show (also by quantitation of colocalization) that glycogen structures induced either by ER stress, Stbd1- or PTG- overexpression do not colocalize with Lamp1. Images featuring Lamp1 immunostaining have been incorporated in revised Fig. 1C, Fig. 5A, Fig. 6A and Fig. S1A.
We further revised the interpretation of the TEM images as recommended by the Reviewer:
o Lines 121-122: "At the ultrastructural level TM-treated cells displayed large intracytoplasmic, not membrane-enclosed, glycogen structures (Fig. 1D-E), not present in control cells (Fig. 1F)."

2. "It is unclear whether the difference between the effects of N-glycosylation inhibitors (TM and 2DG) and the SERCA (Ca-flux) inhibitor (TG) is due to their effects related to ER stress (as presumed by the authors) or due to their primary mechanisms of action listed above. Note that the ER stress is their secondary rather than primary effect. Therefore, selective effects of N-glycosylation inhibitors cannot be interpreted as ER stress effects without demonstrating similar action of unrelated agents inducing ER stress (e.g, Brefeldin A, DTT, etc). An alternate approach is to limit the scope of the paper to tunicamycin-induced effects rather than ER stress effects."
We greatly value the above remark made by the Reviewer. The inhibitors used (TM, TG and 2-DG) are well established inducers of ER stress and in particular TM and TG have been used as ER stressors in numerous studies. Nevertheless, we have addressed the above point raised by the Reviewer and made several experiments with DTT (different concentrations and treatment times). Unfortunately, DTT appeared to have only a very minor effect in inducing glycogen structures as compared to TM, TG or 2-DG. This could be due to different reasons:
a) We may have not determined the optimal concentration and incubation time for the formation of glycogen clusters in C2C12 myoblasts
b) From Oslowski, C. M. and Urano, F. Methods Enzymol. (2011), 490:71-92
"DTT is a strong reducing agent and blocks disulfide-bond formation, quickly leading to ER stress within minutes. Because DTT also blocks disulfide-bond formation of newly synthesized proteins in the cytosol, it is not a specific ER stress inducer."
It is known that Stbd1 dimerizes through its C-terminal domain. It is further thought that this dimerization results in the reorganization of ER membranes and the formation of organized smooth ER structures (OSER) (Demetriadou et. al., J. Cell Sci. (2017), 130:903-915). It could therefore be possible that DTT interferes with Stbd1 dimerization. In such a scenario, although ER stress would be induced by DTT, Stbd1 would fail to induce ER reorganization and therefore glycogen would not be visible as clusters.
We believe that our statement that formation of glycogen clusters in C2C12 myoblasts is an ER stress-induced effect is supported by the provided data:
a) We demonstrate formation of glycogen structures using three different, widely used ER stress inducers which act through different mechanisms
b) We have repeated key experiments performed with TM (inhibitor of N-linked glycosylation) using TG as ER stressor (inhibitor of calcium transport into the ER) and obtained similar findings. These new data are shown in revised Fig. S3 and include
i. Lack of ER stress-induced glycogen structures in shScr myoblasts (Fig.S3A)
ii. Lack of ER stress-induced glycogen structures in the presence of the PERK inhibitor GSK2606414 (Fig. S3B)
iii. Resolution of ER stress-induced glycogen under conditions of glucose starvation (Fig. S3C)
iv. Increased levels of the activated/cleaved Caspase 3 in TG treated shStbd1 cells (Fig. S3D)
3. "It is unclear why the authors switch from myoblasts to MEFs for PTG experiments. With such effective shRNA Stbd1 KD, experiments in cells previously characterized (i.e. C2C12 myoblast cell line or primary myoblasts) should be provided with some interpretation to justify the need for the new cell line. In that case, the new cell line (MEFs) would have to be fully characterized with: (a) IF of GS1, GN and glycogen; (b) glycogen levels; (c) α-amylase degradation; (d) Stbd1-overexpression; and (e) all additional experiments that classify these aggregates as ER-associated or cytosolic."
The Reviewer's remark is correct. We have therefore replaced the results of the PTG experiments performed in MEFs, included in the initial version of the manuscript, with new results obtained in C2C12 cells. The data are shown in revised Fig. 6
4. "It is unclear whether PTG levels also increase with TM or Stbd1 overexpression, whether the glycogen aggregates induced by PTG also colocalize with GS1/GN, or whether these aggregates

© 2020. Published by The Company of Biologists under the terms of the Creative Commons Attribution License (https://creativecommons.org/licenses/by/4.0/).

are in lysosomes. An alternate approach (for both concern #3 and #4) is to limit the scope of the paper to ER-associated glycogen aggregates induced by TM/Stbd1-overexpression in myoblasts."
We thank the Reviewer for the above insightful suggestions. We have addressed the above points as follows:
a. We show by means of real time PCR that PTG (Ppp1r3c) expression levels are not significantly affected by TM treatment or Stbd1 overexpression (revised Fig. 6C)
b. We demonstrate that PTG-induced structures do not colocalize with GS1 but stain weakly positive for GN (revised Fig. 6A)
c. We show by means of Lamp1 immunostaining and quantification of colocalization (thresholded Manders' colocalization coefficient) that PTG-induced structures do not colocalize with lysosomes (revised Fig. 6A)

5. "It is unclear how the authors can assess impacts related to inhibition of IRE1, ATF6 and PERK without proper readouts to confirm activity and specificity of the corresponding inhibitors in their cells under the conditions of their experiments. I recommend either additional experiments or much more cautious discussion of the effects of the inhibitors."
We agree with the Reviewer and are thankful for this remark. We provide data on the action of the inhibitors used on the expression of representative UPR target genes (spliced Xbp1, Atf4, BiP and Chop), assessed by means of real time PCR. These results are shown in revised Fig. 4A.
Minor comments and suggested revisions
a. "The manuscript would be strengthened by the presentation of a cohesive model and some introduction of glycogen and glycogen granules, how glycogen granules differ (if they do) from these larger glycogen clusters, and the mechanisms of glycogenesis and glyocogenolysis."
We have included a paragraph in the Introduction (lines: 40-52) describing the structure of glycogen, glycogen particles and the process of glycogen synthesis and degradation, as suggested by the Reviewer.
b. "WB quantifications would be useful."
We provide densitometry measurements for all western blots in the study in the revised figures, as suggested by the Reviewer.
c. "If available, for consistency, please include intracellular glycogen levels for all experiments to separate glycogen cluster formation/breakdown from glycogenesis/glycogenolysis."
We appreciate the Reviewer's suggestion. However, we have performed quantification of glycogen levels to address specific questions with regards to the role of Stbd1 and don't have quantitative glycogen values available for all experiments. Unfortunately, we cannot provide these values in the revised version.
d. "The manuscript would be strengthened by quantification of glycogen clusters (i.e average number of clusters/cell) and the change with condition."
We provide quantification of the average number of glycogen structures per cell induced by TM, 2-DG and TG. These data are shown in revised Fig. S1B.

e. "The glycogen clusters are referred to as structures, clusters, aggregates, ER aggregates, inclusions, granules and assemblies. As the use of some of these terms may indicate a particular biochemical composition (i.e. aggregates), perhaps limit the variable terms to structures or clusters. The term 'ER aggregates' is especially confusing as it traditionally refers to protein aggregates that form within the ER lumen."
We agree with the Reviewer that the use of different terms to describe the same cellular structure may cause confusion. As recommended by the Reviewer we have replaced all other definitions with the terms "structures" and "clusters" which are used throughout the text, starting with the title.
f. "In Fig. 1, the authors characterize the TEM images with the claim "TM treatment induced the formation of glycogen-containing ER aggregates (F, arrow). Representative examples of membrane-associated glycogen granules are shown by arrowheads in G…" It is unclear how the TEM displays membrane-associated glycogen assemblies. How are these being characterized?"
We acknowledge that the Reviewer is right and have therefore removed the above sentence.
g. "The interpretation of Fig. 1 E is questionable. In the TEM image, the cell appears extremely unhealthy, with large amounts of blebbing, making it very difficult to resolve organelles (especially ER). As a fixative composed of 2.5%gluteraldehyde in 0.1M phosphate buffer instead of the more traditional 0.1M sodium cacodylate was used, I am unsure whether the indicated regions of dilated ER are in fact dilated ER, or simply ruptured organelles (e.g. mitochondria) or another fixation-related artifact."

© 2020. Published by The Company of Biologists under the terms of the Creative Commons Attribution License (https://creativecommons.org/licenses/by/4.0/).

We agree with the Reviewer that the cell shown in Fig. 1E (initial version of the manuscript) appears unhealthy. However, we can exclude a fixation-related artefact since both TM-treated cells and DMSO-treated controls (which appear normal) were fixed in parallel in the same fixative and for the same time. However, the Reviewer is correct that the statement about ER dilation based on the image cannot be safely made and we have therefore removed the image from the panel in revised Fig. 1.
h. "Please provide associated calnexin stain for Figure 6C, if this experiment is retained in the revised manuscript."
Results shown in Fig. 6 (initial version of the manuscript) performed in MEFs have been replaced with experiments in C2C12 myoblasts. As requested by the Reviewer we provide calnexin staining in shScr and shStbd1 C2C12 myoblasts in revised Fig. 6B.

i. "It is unclear why Chop nuclear translocation was used to indicate ER stress induced by TM in MEFs (Fig. S3), but an increase in BiP was used elsewhere (Figure 4)."
Fig. 6 has been revised according to the Reviewer's suggestion. The revised Fig. 6 shows data obtained in C2C12 myoblasts. Accordingly, images shown in Fig. S3 (initial version of the manuscript) have been removed.

Second decision letter

MS ID#: JOCES/2020/244855

MS TITLE: Stbd1 promotes glycogen clustering during endoplasmic reticulum stress and supports survival of mouse myoblasts

AUTHORS: Andria A. Lytridou, Anthi Demetriadou, Melina Christou, Louiza Potamiti, Nikolas P Mastroyiannopoulos, Kyriacos Kyriacou, Leonidas A Phylactou, Anthi Drousiotou, and Petros P Petrou
ARTICLE TYPE: Research Article

I am happy to tell you that your manuscript has been accepted for publication in Journal of Cell Science, pending standard ethics checks. In making your final checks, please consider the minor suggestions regarding your figure legends by reviewer #2.

Reviewer 1

Advance summary and potential significance to field

A demonstration that ER stress induces the expression of glycogen-binding protein Stbd1 in mouse myoblasts to promote glycogen aggregation.

Interestingly, these glycogen aggregates are degraded under glucose depleted conditions in autophagy/lysosome-independent manner.

-failure to induce glycogen aggregation during ER stress, as is the case in Stbd1 knockdown cells, results in increased apoptosis.

- ER stress induces Stbd1 expression and that Stbd1 is required for the formation of glycogen aggregates

Comments for the author

The Authors have improved their study and addressed my suggestions adequately

© 2020. Published by The Company of Biologists under the terms of the Creative Commons Attribution License (https://creativecommons.org/licenses/by/4.0/).

Reviewer 2

Advance summary and potential significance to field

In this manuscript, these authors report that myoblasts, in response to treatment with inducers of ER stress, have significantly increased levels of Stbd1 and glycogen cluster formation. This upregulation of Stbd1 occurs specifically through the PERK arm of UPR. Furthermore, these clusters dissociate through an autophagy-independent mechanism.

Stbd1-mediated formation of ER-associated glycogen clusters in response to ER stress could have far-reaching implications with regards to the relationship between ER stress and metabolism, which is likely critical during periods of myoblast differentiation and skeletal muscle development; but with the potential for a broader impact on other tissues

Comments for the author

The authors have adequately addressed my concerns for this manuscript.

I have a few minor comments that I leave as suggestions rather than revisions.

a - Figure 4A and C legend was slightly confusing. Perhaps for clarity, adjust the legends to: Untreated, TM, TM + 4u8c, TM + AEBSF, TM + GSK2606414

b - In Figure 6, the figure could benefit with a label indicating PTG-Myc was transfected. Perhaps it could be added to the top as it was in Figure 5, but with 'C2C12 + PTG-Myc' instead of the C2C12/Stbd1 used to demarcate stable lines.

© 2020. Published by The Company of Biologists under the terms of the Creative Commons Attribution License (https://creativecommons.org/licenses/by/4.0/).
